# The in situ generation and reactive quench of diazonium compounds in the synthesis of azo compounds in microreactors

**DOI:** 10.3762/bjoc.12.186

**Published:** 2016-09-06

**Authors:** Faith M Akwi, Paul Watts

**Affiliations:** 1 Nelson Mandela Metropolitan University, University Way, Port Elizabeth, 6031, South Africa

**Keywords:** azo coupling, diazotization, microreactor, scale up

## Abstract

In this paper, a micro-fluidic optimized process for the continuous flow synthesis of azo compounds is presented. The continuous flow synthesis of Sudan II azo dye was used as a model reaction for the study. At found optimal azo coupling reaction temperature and pH an investigation of the optimum flow rates of the reactants for the diazotization and azo coupling reactions in Little Things Factory-MS microreactors was performed. A conversion of 98% was achieved in approximately 2.4 minutes and a small library of azo compounds was thus generated under these reaction conditions from couplers with aminated or hydroxylated aromatic systems. The scaled up synthesis of these compounds in PTFE tubing (i.d. 1.5 mm) was also investigated, where good reaction conversions ranging between 66–91% were attained.

## Introduction

Going green, a familiar catch phrase in the chemical industry, in addition to environment protection laws have influenced and also triggered the development of cleaner methods of production. The production of azo compounds is one controversial sector of the fine chemical industry; color is highly desired and used in almost everything, but the waste generated from the production of these compounds is detrimental to the environment and human health.

Following the principles of green chemistry [1–2] such as less hazardous chemical synthesis, efficient atom economy, reduction of waste produced, some alternative cleaner methods for the synthesis of azo dyes have been developed. These methods are however only representative of particular coupling agents and diazotized amines. Nonetheless they highlight the green benefits that they offer.

**Scheme 1:**
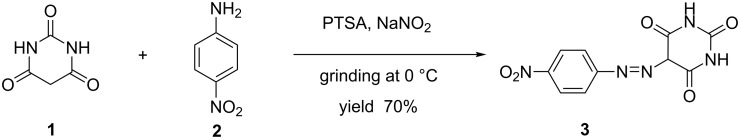
PTSA-catalyzed diazotization and azo coupling reaction.

For example Noroozi-Pesyan et al. synthesized azo dyes by grinding derivatives of aniline with solid sodium nitrite in the presence of *p*-toluenesulfonic acid [3]. It was found that the yield of isolated azo dyes obtained increased with an increase in electron donor strength of the coupling compound. This method eliminates the use of alkaline and acidic solutions (Scheme 1).

**Scheme 2:**
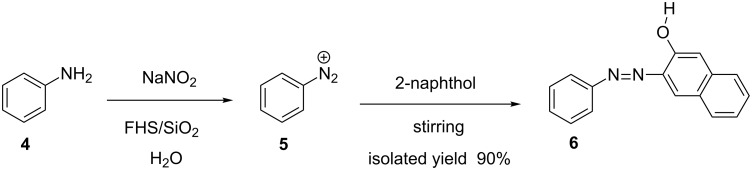
Ferric hydrogen sulfate (FHS) catalyzed azo compound synthesis.

In another method developed by Rahimizadeh et al., ferric hydrogen sulfate was used as a catalyst to synthesize azo compounds from aromatic amines and 2-naphthol. The method boasts of shorter reaction times [4] with high yields (Scheme 2).

**Scheme 3:**
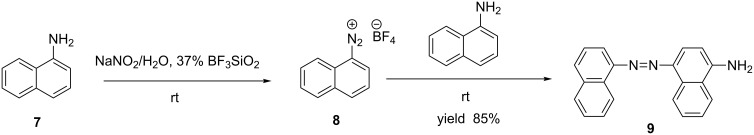
Synthesis of azo compounds in the presence of silica supported boron trifluoride.

Mirjalili et al. also used silica supported boron trifluoride and was able to carry out diazotization at room temperature [5] after they discovered that the diazonium salts obtained were stable at room temperature even in their dry state. In addition, the method also facilitated short reaction times and provided high yields (Scheme 3).

A number of other methods have been developed for the green synthesis of azo compounds [6–16]. Thus far, the above examples concentrate on modifying the procedure of diazotization and azo coupling (using compounds that impart green benefits to the process or make it environment friendly, i.e., short reaction times thus less energy is required for the process, high yield thus low amount of waste generated, etc). Equally, process equipment can also be changed or modified to achieve the above mentioned green benefits in addition to other advantages.

For example, isolated diazonium salts are known to be hazardous due to their explosive and unstable nature. However, microreactor technology makes it possible to safely perform reactions with unstable intermediates [17] such as these, as well as those that give rise to explosive [18] and hazardous products [19]. The small reagent volumes used in microreactors also reduce the amount of acidic and alkaline waste associated with the synthesis of azo compounds during research and development.

In the conventional way of performing reactions, the amount of waste generated is dealt with at the end of the reaction. On the contrary, microreactor technology enables the reduction of waste generated by increasing the atom efficiency of reactions and in so doing, the quantity of starting materials is reduced in turn minimizing the amount of waste generated. This aspect of microreactor technology will definitely prove to be quite important in the synthesis of these compounds more so that their production, however important they are, has adverse effects on the environment, mammals [20–21] and aquatic life. Even with a number of dye degradation techniques [22] currently being employed in waste water treatment, a certain percentage of the dyestuffs is still found in water bodies. This therefore is motivation for developing better methods or techniques or processes that can be used independently or in conjunction with the existing techniques. Microreactor technology is one such technology that can be used in the manufacture of these dyes. If used in conjunction with existing azo dye degradation techniques the amount of waste generated can easily be managed.

**Scheme 4:**
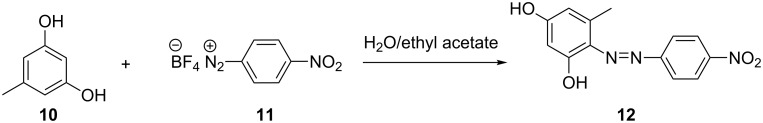
Phase transfer catalyzed azo coupling of 5-methylresorcinol in microreactors.

Hisamoto et al., for example, used ‘phase transfer synthesis’ in micro-chips for a diazo-coupling reaction [23]. The authors did not however employ a phase transfer catalyst, but rather the principle to increase the reaction selectivity in the diazo coupling of 5-methylresorcinol (**10**) to *p*-nitrobenzene diazonium tetrafloroborate (**11**) in a biphasic laminar flow reaction system. The bi-phasic reaction media consisted of compound **10** dissolved in the organic phase, ethyl acetate (C_4_H_8_O_2_) and **11** in the aqueous phase (Scheme 4). The large specific interfacial areas and reduced molecular diffusion distances were found to have played a role in avoiding the undesirable side reaction, thus increasing the atom economy in the reaction. This in turn reduces the amount of waste generated after the reaction. A reaction conversion of almost 100% was attained in 2.3 seconds. The same reaction performed at a macro scale and at a strong stirring rate providing a calculated specific interfacial area of 40 cm^2^, gave a comparable conversion to that attained at a micro scale (calculated specific interfacial area of 80 cm^2^).

In the synthesis of azo dyes and pigments, the cost of production and quality of the product cannot be over looked. Wille et al., in their investigative research involving the synthesis of two azo pigments (yellow and red pigments) in microreactors [24], demonstrated that scaling out in the microreactors provided better and more consistent quality of the pigments as compared to scale up in the batch vessels.

**Scheme 5:**
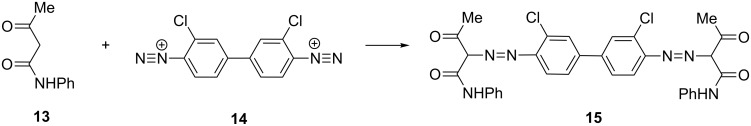
Synthesis of yellow pigment 12 in a micro-mixer apparatus.

Similarly, yellow pigment 12 (**15**) was also synthesized by Pennemann et al. (Scheme 5) using a micro-mixer apparatus [25]; the group’s comparison of the results with the batch synthesis of the said pigment **15** affirmed the notion that mixing is an essential unit operation in the synthesis of azo pigments.

The pigment synthesized in a micro-mixer (25 µm channel width) at a flow rate of 30 mL/min had smaller pigment size distribution compared to the batch synthesized pigment. The fast mixing in the micro-mixer was noted to be responsible for the improvement of glossiness (73%) and tinctorial strength (66%) of the yellow pigment thus yielding a good quality product.

With all the various applications of azo compounds previously mentioned, it is therefore important to develop an optimized process for their synthesis. This was achieved with the use of the microreactor technology. Since the benefits of microreactor technology are well documented in literature [26–30], the ease of reaction parameter optimization in the synthesis of azo compounds in microreactors is highlighted.

**Scheme 6:**
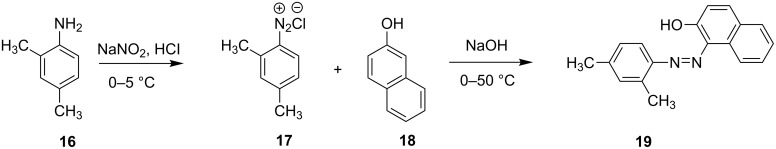
Continuous flow synthesis of Sudan II azo dye in LTF-MS microreactors.

In this study, the continuous flow synthesis of Sudan II azo dye (**19**, Scheme 6) constituting of the diazotization of 2,4-dimethylaniline (**16**) and its in situ azo coupling to 2-naphthol (**18**) within LTF-MS microreactors was investigated.

Although various groups have investigated similar reactions in microreactors, to the best of our knowledge, there is no detailed study combining the effect of pH, temperature and flow rate on the azo coupling reaction. In addition, industry always questions why reactions are done in micro structured reactors rather than simple tubular reactors. As such, we extended this investigation to study the optimized conditions obtained within the LTF microreactors within PTFE tubing (i.d. 1.5 mm) in order to scale up the synthesis and to see if the size effect had any major implication on the reaction performance.

## Results and Discussion

### Azo coupling reaction in the synthesis of 1-((2,4-dimethylphenyl)azo)naphthalen-2-ol in LTF-MS microreactors

In an effort to exemplify the azo coupling of phenols as well as naphthol derivatives in alkaline reaction conditions, the synthesis of 1-((2,4-dimethylphenyl)azo)naphthalen-2-ol (**19**) also commonly known as Sudan II azo dye was used as a model reaction. The synthesis involved diazotization of 2,4-dimethylaniline (**16**) to form diazonium salt intermediate **17**, which is coupled with 2-naphthol (**18**) under alkaline conditions (Scheme 6). The experimental set up comprised of two syringes, two syringe pumps and an LTF-MS microreactor placed into a temperature control bath (as shown in Figure 6 in the experimental section). Following the experimental procedures outlined, a range of reaction conditions were evaluated, where the pH value is that of the stock solution (Table 1).

**Table 1:** Data showing conversions attained at different reaction conditions.

Run	pH	Flow rate (mL/min)	Temperature (°C)	Conversion (%)

1	5.11	0.6	7	59
2	5.11	0.37	26	62
3	5.11	0.03	25	64
4	5.11	0.13	40	63
5	5.11	0.37	46	57
6	5.11	0.7	26	55
7	5.11	0.6	43	57
8	5.11	0.37	2	54
9	5.11	0.13	8	56
10	5.11	0.37	26	56
11	7.39	0.6	7	70
12	7.39	0.37	26	78
13	7.39	0.13	41	76
14	7.39	0.37	49	73
15	7.39	0.7	25	76
16	7.39	0.6	43	72
17	7.39	0.37	2	74
18	7.39	0.13	8	82
19	7.39	0.37	26	77
20	10.83	0.37	26	72
21	10.83	0.13	40	69
22	10.83	0.37	50	69
23	10.83	0.7	26	73
24	10.83	0.6	43	71
25	10.83	0.37	26	64

A quadratic model was fitted onto the resultant calculated conversions of 2-naphthol as obtained from reversed-phase HPLC analysis output. STATISCA 12 – Statsoft program was thereafter used to validate the model fitted. The findings of this investigation are presented here in the form of simple profile plots.

### Effect of reaction temperature, flow rate and pH on the reaction conversion

The pH is of utmost importance in the azo coupling reaction, the second step in the synthesis of azo compounds. This reaction parameter is also dependent on the kind of coupling compound used [31]. For example, phenols are successfully coupled in alkaline conditions in which the phenolate ion is formed. These conditions provide the desired electron-releasing group thus facilitating the electrophilic substitution reaction to afford the azo compound. However, highly alkaline conditions are usually avoided as they lead to diazonium salt decomposition.

In the regression summary of the statistical data analysis (Table 2) of the observed data, it is seen from the *p*-values (0.067 and 0.084) associated with the estimated coefficients (b) of flow rate and temperature respectively, that these two reaction parameters seem not to have a significant effect on the reaction conversion in the experimental domain employed for this investigation. In contrast, pH is shown to be of importance in this reaction considering that the *p*-value associated with its estimated coefficient (b) is less than 0.005. Furthermore, evidence of a quadratic relationship between pH and reaction conversion was also seen (pH^2, *p* < 0.005). Similarly, there was also a slight indication of a quadratic relationship between temperature and the response (Temperature^2, *p* = 0.076). There were no interactions observed between the reaction parameters investigated.

**Table 2:** Statistical multiple regression analysis output.

N = 25	Regression summary for dependent variable: conversion %					

	b*	Std. Err. (of b*)	b	Std. Err. (of b)	t(19)	*p*-value

Intercept			−42.4512	10.97819	−3.86687	0.001038
pH	7.54901	0.772030	27.9443	2.85784	9.77812	0.000000
Flow rate	−0.15896	0.081676	-6.5854	3.38369	−1.94621	0.066572
Temperature	0.57407	0.315099	0.3187	0.17495	1.82187	0.084257
pH^2	−7.02309	0.774875	−1.6268	0.17949	−9.06351	0.000000
Temperature^2	−0.58488	0.311906	−0.0061	0.00325	−1.87518	0.076223

**Figure 1:**
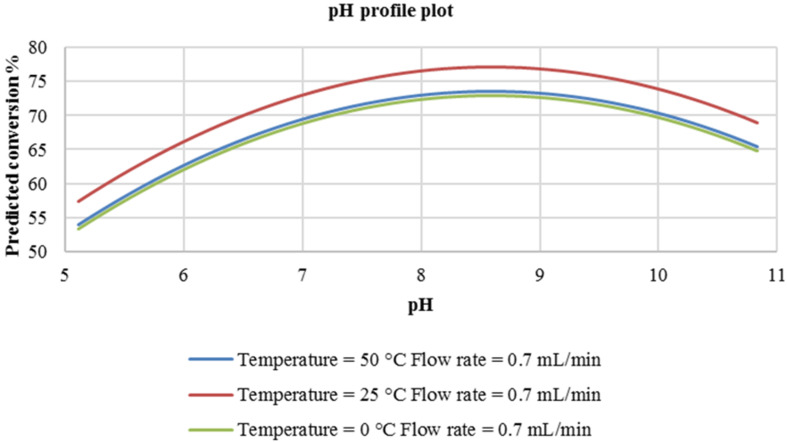
pH profile plot at constant flow rate of 0.03 mL/min.

In the plot (Figure 1), it is seen that at a flow rate of 0.03 mL/min, the pH has a non-linear relationship with the predicted conversion of the coupler. In addition, the predicted conversion increases with an increase in reaction pH in this experimental domain. The optimum pH that would provide the best conversion was predicted to be approximately 8.5 and the most suitable reaction temperature was found to be 25 °C. Therefore, at a pH of about 8.5 and a reaction temperature of 25 °C, a reaction conversion of approximately 80% should be attained. As expected, at highly alkaline conditions (pH 9–11), the reaction conversion is predicted to gradually decrease. It is also seen that at 0 °C and 50 °C, slightly lower conversion is predicted (approximately 74%–75%). This could be attributed to decomposition of the reaction intermediate at higher temperatures (50 °C) and lowered rate of reaction at lower temperatures (0 °C).

**Figure 2:**
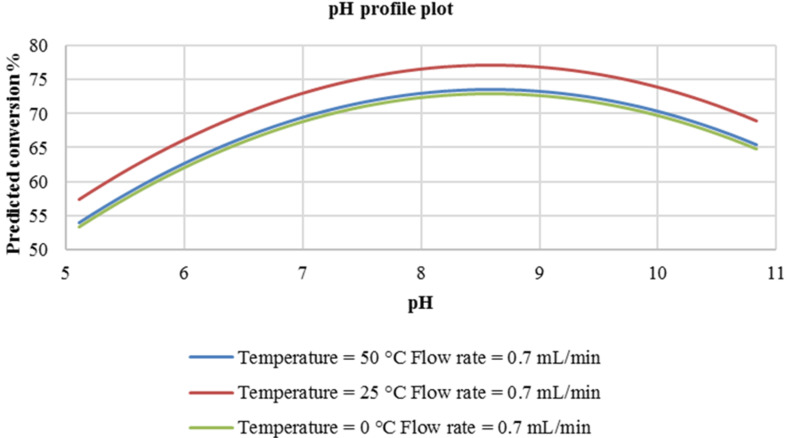
pH profile plot at a constant flow rate of 0.7 mL/min.

At a higher flow rate of 0.7 mL/min (Figure 2), a similar trend is observed with regard to the effect of pH on the reaction conversion. Similarly, a reaction temperature of 25 °C was predicted to provide optimum conversion (75%). At this reaction temperature (25 °C), the predicted reaction conversion at this flow rate, is slightly lower (75%) compared to that predicted at a flow rate of 0.03 mL/min (80%). It was then concluded that the flow rate of the reactants in this experimental domain had no significant effect on the reaction response. For information, this flow rate range covers a residence time of 0.37 to 0.86 minutes.

### Azo coupling reaction in the synthesis of 4-(2-(4-nitrophenyl)diazenyl)-*N*-phenylbenzenamine in LTF-MS microreactors

The synthesis of azo compounds involving couplers containing aminated aromatic systems is usually carried out in slightly acidic reaction conditions. The synthesis of 4-(2-(4-nitrophenyl)diazenyl)-*N*-phenylbenzenamine was an interesting choice for demonstrating the synthesis of azo compounds involving couplers containing aminated aromatic systems.

**Scheme 7:**
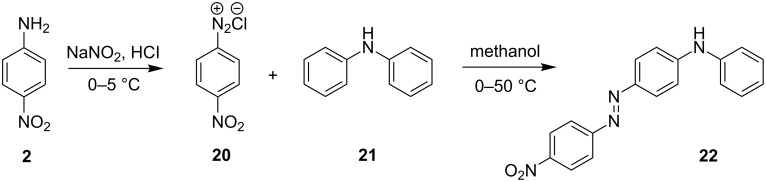
Azo coupling reaction under acidic conditions.

It involves the diazotization of *p*-nitroaniline **2** to form **20** which is subsequently coupled to **21**. Diphenylamine (**21**), the coupler used in this reaction is sparingly soluble in aqueous media thus rendering it quite difficult to use in this synthesis and as such, methanol was used as the azo coupling reaction media (Scheme 7). It is due to this that the pH of the diazonium compound solution was buffered to the preferred values for the reaction investigation as opposed to the conventional buffering of the coupler solution. Following the experimental procedures, a range of reaction conditions were evaluated (Table 3).

**Table 3:** Data showing the conversion attained at various reaction conditions.

Run	pH	Flow rate (mL/min)	Temperature (°C)	Conversion (%)

1	3.5	0.6	6	84
2	3.5	0.37	26	89
3	3.5	0.13	41	85
4	3.5	0.37	47	83
5	3.5	0.7	26	78
6	3.5	0.6	41	83
7	3.5	0.37	3	84
8	3.5	0.13	7	83
9	3.5	0.37	26	83
10	5.66	0.6	8	92
11	5.66	0.37	26	93
12	5.66	0.13	43	91
13	5.66	0.37	50	90
14	5.66	0.7	25	96
15	5.66	0.6	42	86
16	5.66	0.37	2	97
17	5.66	0.13	7	96
18	5.66	0.37	26	99
19	6.94	0.6	7	81
20	6.94	0.37	25	74
21	6.94	0.13	8	86
22	6.94	0.7	43	70
23	6.94	0.13	2	86
24	6.94	0.37	26	56
25	6.94	0.37	7	58
26	6.94	0.6	50	64
27	6.94	0.37	43	86

A Logit model was then fitted onto the resultant calculated conversions of diphenylamine as obtained from reversed phase HPLC analysis output. STATISCA 12 – Statsoft program was thereafter used to validate the model fitted (see Supporting Information File 1). The result of this investigation is also presented here in form of simple profile plots.

### Effect of reaction pH, temperature and flow rate on the reaction conversion

Azo coupling reactions involving aromatic amines as coupling agents are carried out in mildly acidic conditions such that a water-soluble protonated version of the aromatic amine, which is more reactive than its unprotonated version is availed; it is also obvious that the protonation also renders the aromatic ring less nucleophilic.

In this reaction, like the one previously discussed, pH plays an important role and significantly affects the reaction conversion (*p* < 0.005) moreover (Table 4), it also has a non-linear effect on this response (pH^2, *p* < 0.005). Conversely, within this experimental domain, there is evidence (Flow rate, *p* = 0.116) that shows that the flow rate of the reactants has a negligible effect on the reaction conversion. Looking at the *p*-value associated with the estimated coefficient (b) of the reaction temperature (temperature, *p* = 0.012), there is a slight indication of its significance on the reaction conversion.

**Table 4:** Statistical multiple regression analysis output.

N = 24	Regression summary for dependent variable: logit					

	b*	Std. Err. (of b*)	b	Std. Err. (of b)	t(19)	*p*-value

Intercept			8.14853	1.409724	5.78023	0.000014
pH	−9.27407	1.208038	−4.48258	0.583899	−7.67697	0.000000
Temperature	0.29741	0.107162	0.01204	0.004340	2.77534	0.012053
Flow rate	0.17639	0.107167	0.61041	0.370851	1.64596	0.116214
pH^2	9.30475	1.208036	0.43744	0.056793	7.70238	0.000000

**Figure 3:**
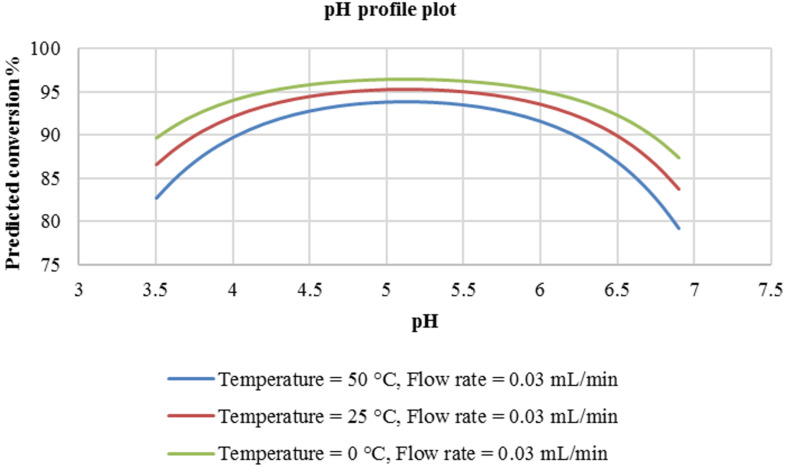
pH profile plot at a constant flow rate of 0.03 mL/min.

It is also demonstrated in Figure 3 that the effect of pH on the azo coupling reaction is quite conspicuous. It is seen that the reaction flourishes at a mildly acidic pH that being between 5.5 and 6.0 while temperature is predicted not to have a tremendous effect on the reaction conversion (Figure 3). A variation in reaction temperature between 0 °C and 50 °C provides a very slight improvement in conversion (from 82% to 95%).

**Figure 4:**
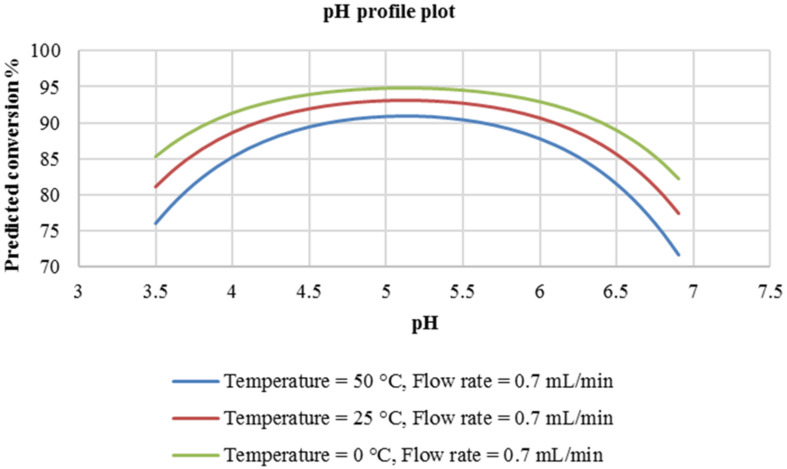
pH profile plot at constant flow rate of 0.7 mL/min.

The flow rate of the reactants (diazotized primary amine and coupler) was also investigated as mentioned earlier on. As is shown in Figure 4, based on the predicted conversion, there is no difference in carrying out the reaction at either 0.03 mL/min or 0.7 mL/min.

**Figure 5:**
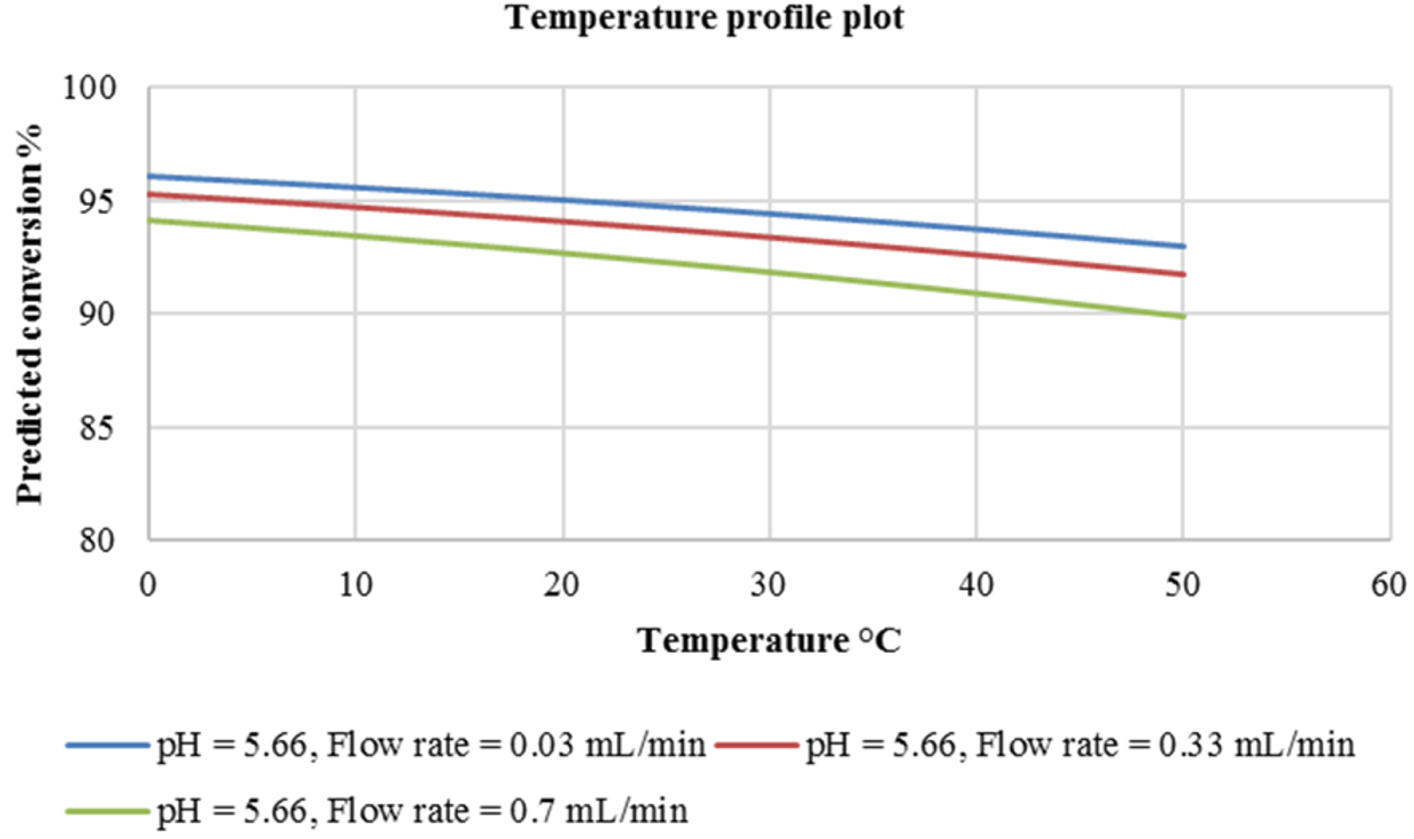
Temperature profile plot at constant pH 5.66.

Moving on to the reaction temperature, unlike pH, it has a more or less linear relationship with predicted reaction conversion. There is a slight drop in the predicted conversion as the reaction temperature is increased from 0 °C to 50 °C. This is clearly shown in Figure 5 below.

An increase in the flow rate from 0.03–0.7 mL/min at a constant pH of 5.66 causes a 2% drop in predicted conversion whereas an increase in the reaction temperature from 0–50 °C at flow rates of 0.03, 0.33 and 0.7 mL/min at constant pH 5.66 is shown to lead to a less than 5% drop in the predicted conversion. It was thereafter concluded that in this experimental domain, the reaction temperature had no significant effect on the reaction conversion. For both reactions investigated thus far, it was found that the flow rate of the reactants in the chosen experimental domain had little or no significant effect on the reaction conversion.

The effect of reaction parameters i.e. flow rate, temperature and pH on azo coupling reactions in the synthesis of azo compounds under acidic and alkaline reaction conditions in LTF-MS microreactors however, was successfully demonstrated. We therefore went ahead to fully make good use of the benefits that microreactor technology offers to organic syntheses such as this one by performing both reaction steps in continuous flow reactors.

### Continuous flow synthesis of Sudan II azo dye in LTF-MS microreactors

Having determined the reaction parameters that affect the azo coupling reaction in the synthesis of Sudan II azo dye, an attempt to perform both reaction steps involved in this synthesis in continuous flow reactors was thus made. This was achieved in LTF-MS reactors with the aid of statistical modeling where the continuous flow synthesis of Sudan II azo dye was optimized and used a model reaction.

Based on the statistical experimental central composite design used for the optimization of this synthesis, no descriptive trends showing the effect of the flow rates of reactants on the conversion of 2-naphthol could be obtained. At all varied reaction parameters in the 20 experiments carried out, the response was relatively the same with no clear cut trends observed as is shown in Table 5. A quadratic regression model was then fitted onto the observed data and no outliers were found during this model fitting procedure in addition, there were also no indications of either synergistic or antagonist interactions between the independent variables (see Supporting Information File 1). On further statistical analysis of this data, it was found that there is some evidence that supports the notion that the flow rate of the (amine + HCl) solution in this particular experimental domain has the most effect on the conversion (Partial correlation: 0.406, *p* = 0.094). The R-square value corresponding to the three variables investigated was also found to be nil which indicated that there was no correlation between the variables investigated.

**Table 5:** Data showing conversions attained at various reaction conditions.

Run	Amine + HCl (mL/min)^a^	Sodium nitrite (mL/min)^b^	Coupler (mL/min)^c^	Conversion (%)

1	0.20	0.01	0.07	98
2	0.09	0.03	0.07	97
3	0.30	0.03	0.07	97
4	0.26	0.02	0.03	99
5^d^	0.20	0.03	0.07	98
6	0.26	0.04	0.03	99
7	0.13	0.02	0.10	93
8	0.26	0.04	0.10	98
9^d^	0.20	0.03	0.07	98
10	0.13	0.04	0.10	96
11	0.20	0.05	0.07	96
12	0.20	0.03	0.12	95
13	0.20	0.03	0.01	96
14^d^	0.20	0.03	0.07	95
15	0.13	0.04	0.03	96
16^d^	0.20	0.03	0.07	95
17^d^	0.20	0.03	0.07	95
18	0.13	0.02	0.03	98
19^d^	0.20	0.03	0.07	98
20	0.26	0.02	0.10	97

^a^Amine-flow rate of HCl + amine (2,4-dimethylaniline) solution, ^b^nitrite-flow rate of sodium nitrite solution, ^c^coupler-flow rate of coupler (2-naphthol) solution. ^d^center point.

The reaction conditions at the center point (run with letter 'd' in the Table 5 above) of the central composite design used for the optimization were therefore used to generate a small library of compounds. Before embarking on this task, a confirmatory experiment to ascertain the reproducibility of the reaction output at these reaction conditions was performed and indeed a similar result was obtained (Table 6).

**Table 6:** Confirmatory experiment for the chosen reaction parameters for the synthesis.

Run	Amine + HCl (mL/min)^a^	Sodium nitrite (mL/min)^b^	Coupler (mL/min)^c^	Conversion (%)

**1**	0.2	0.03	0.07	98
**2**	0.2	0.03	0.07	98

^a^Amine-flow rate of HCl + amine (2,4-dimethylaniline) solution, ^b^nitrite-flow rate of sodium nitrite solution, ^c^coupler-flow rate of coupler (2-naphthol) solution.

### The continuous flow synthesis of 2-naphtholic, phenolic and similar azo compounds in LTF-MS microreactors

At the reactant flow rates stated in the confirmatory experiment in Table 6, similar azo compounds were synthesized. To our delight, the reaction conditions were robust since comparable high conversions were also attained for the synthesis of similar azo compounds regardless of the substituent groups present on the coupler as well as diazotizable amine. This is shown in Table 7.

**Table 7:** Azo compounds synthesized under alkaline and acidic azo coupling conditions.

Run	Diazotizable amine	Coupler	Product	Conversion

1	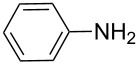	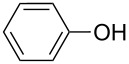	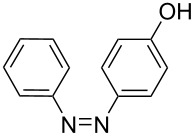	97%
2	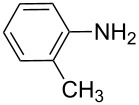	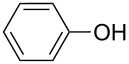	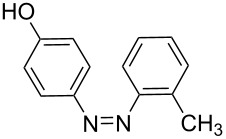	88%
3	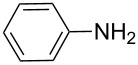	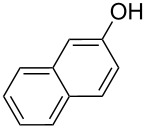	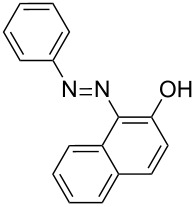	95%
4	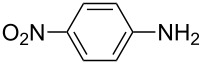	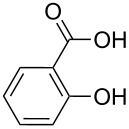	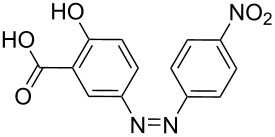	80%
5	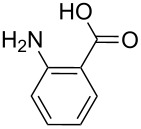	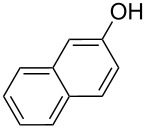	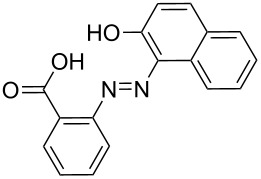	92%
6	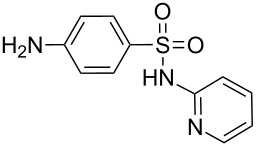	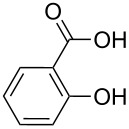	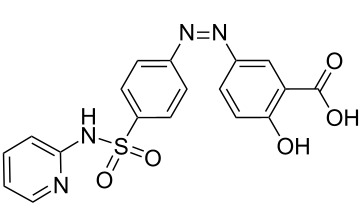	80%
7	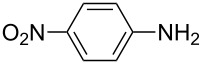	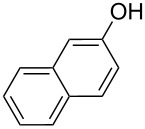	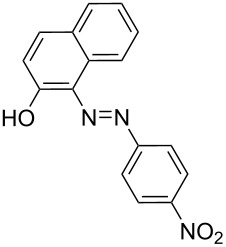	88%
8	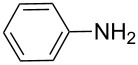	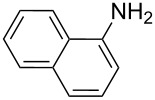	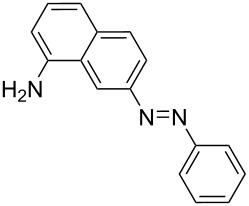	84%
9	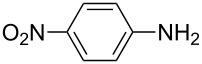	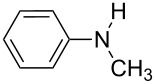	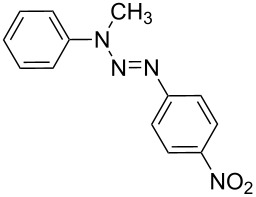	87%
10	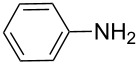	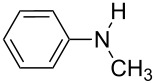	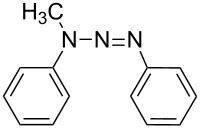	79%
11	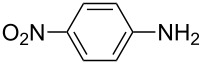	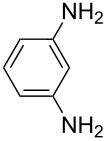	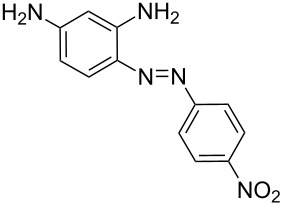	89%
12	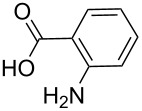	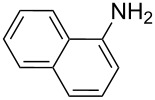	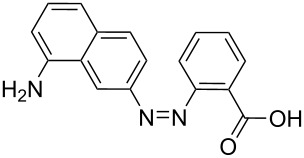	90%
13	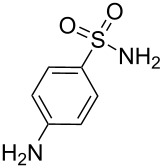	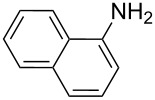	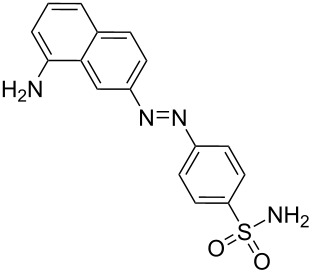	88%
14	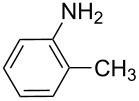	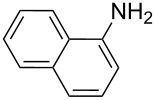	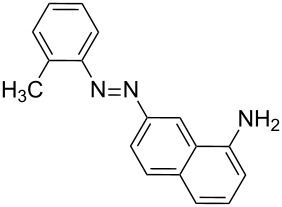	82%

### The continuous flow scaled-up synthesis of 2-naphtholic, phenolic and similar azo compounds in LTF-MS microreactors

In the synthesis of azo compounds, there is usually formation of a precipitate, which renders this reaction problematic in microreactors due to blockages. The geometrical specifications of the microchannel are very important, so much that these dictate the concentration of the reagents used for the reaction, amount of solvent added to facilitate quick dissolution of particulates as they are formed as well as the output of the reaction, i.e., conversion, yield and or even selectivity. The scaled-up synthesis of these compounds in PTFE tubing of 1.5 mm internal diameter is herein reported. The experimental set up of the scaled-up synthesis is shown in the experimental section (Figure 8). A comparison of the two microreactor systems, i.e., LTF-MS and the PTFE tubing microreactor systems is presented in Table 8. The reactions were conducted at appropriate flow rates so as to maintain the same residence time used in the LTF-MS microreactors.

It was found that the synthesis of these compounds in a simple set up comprising of PTFE tubing (i.d. 1.5 mm) provided comparable conversions to those attained in the LTF-MS microreactors despite the geometrical specifications of the two reactor systems being quite different. It should also be noted that the PTFE tubing is much cheaper than the glass LTF-MS and at the internal diameter of the PTFE tubing used there were no occurrences of blockages witnessed due to precipitate formation. This is particularly important since the amount of solvent used in the reactions can be kept at a bare minimum. Despite the fact that the geometrical specifications of the two reactor systems (LTF-MS microreactors and PTFE tubing) were quite different, the drop in conversion is not massive and can be circumvented by increasing the residence time in order to achieve even better conversions.

**Table 8:** Comparison of reaction conversion attained from two continuous flow reactor systems.

Run	Diazotizable amine	Coupler	Product	Conversion	

				LTF-MS (CD: 1.0 mm)	PTFE (i.d.: 1.5 mm)

1	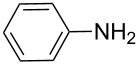	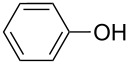	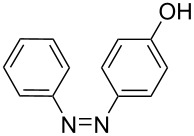	97%	90%
2	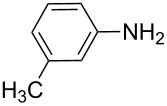	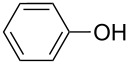	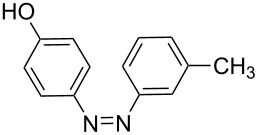	88%	91%
3	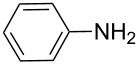	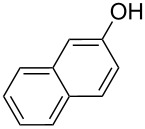	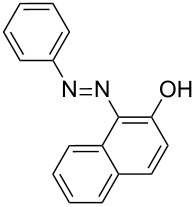	95%	69%
4	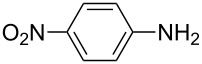	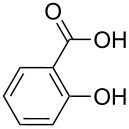	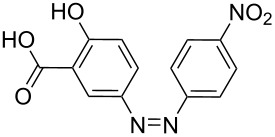	80%	71%
5	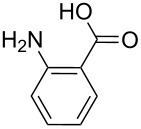	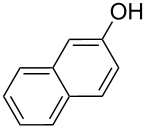	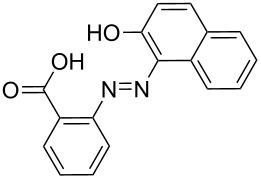	92%	70%
6	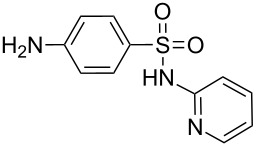	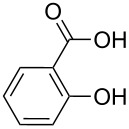	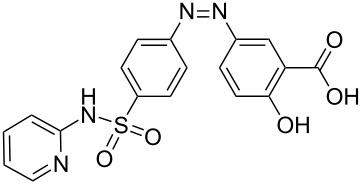	80%	70%
7	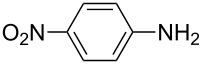	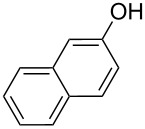	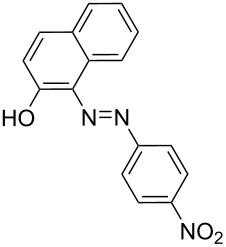	88%	67%
8	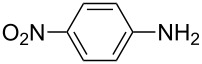	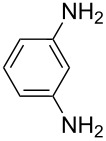	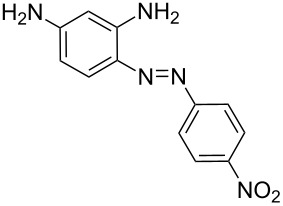	89%	80%
9	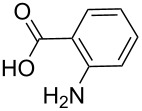	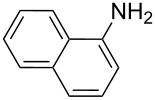	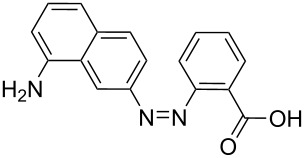	90%	72%
10	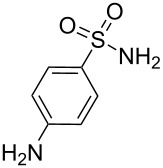	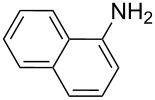	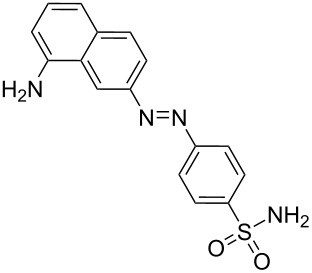	88%	78%
11	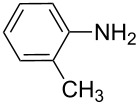	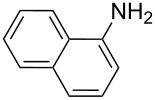	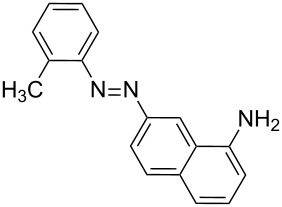	82%	66%

## Conclusion

From our proof of concept investigation, the desired temperature and pH in the azo coupling of hydroxylated or aminated couplers in the synthesis of azo compounds was determined. It was found that at slightly alkaline conditions (pH 8.55) and at a temperature of 25 °C, excellent conversions were attained in the azo coupling reaction of the diazonium salt solution of 2,4-dimethylaniline to 2-naphthol whereas the azo coupling reaction of the diazonium salt solution of *p*-nitroaniline to diphenylamine was found to thrive at a pH of 5.71 and at a temperature of 25 °C. It should be noted that the data generated was obtained in a very short time. On the down side, for a couple of experiments, samples could not be collected due to the limitations of the microreactor used. There were blockages observed due to precipitation of the product in the microreactor channels. This was particularly observed in the synthesis of 1-((2,4-dimethylphenyl)azo)naphthalen-2-ol at slow flow rates, temperatures close to 25 °C and pH greater than 7. Nonetheless, the effect of the reaction parameters on the azo coupling reaction in the synthesis of azo compounds was shown. To the best of our knowledge, this investigation is the first of its kind to expound the effect of pH, flow rate and temperature on the azo coupling reaction in the synthesis of azo compounds. A simple and fast continuous flow process was also thereafter developed for the synthesis of naphtholic, phenolic and similar azo dyes. The robustness of the process was clearly demonstrated. In addition, an easy scale-up strategy was also established where it was found that the synthesis of these compounds in a simple continuous flow set up consisting of T-mixers and PTFE tubing (i.d.: 1.5 mm) provided relatively satisfactory reaction conversions moreover no occurrence of blockages was observed when this set up was in use. This finding is of importance especially when it comes to an increasing reaction throughput by the numbering up technique. Ideally, in evaluating the performance of two reactor systems in a chemical synthesis, it is important to keep most factors constant especially those pertaining to the geometry of the reactor channel as this can affect the reaction output. Albeit comparable reaction conversions were attained from the two reactor systems investigated, it is worth determining the role that the difference in geometrical structure had to play in the data observed in this study.

## Experimental

All chemicals and solvents used were of analytical grade. ^1^H and ^13^C NMR spectra were recorded on a Bruker Avance-400 (400 MHz).

### Microreactor set up; azo coupling reactions in the synthesis of azo compounds

**Figure 6:**
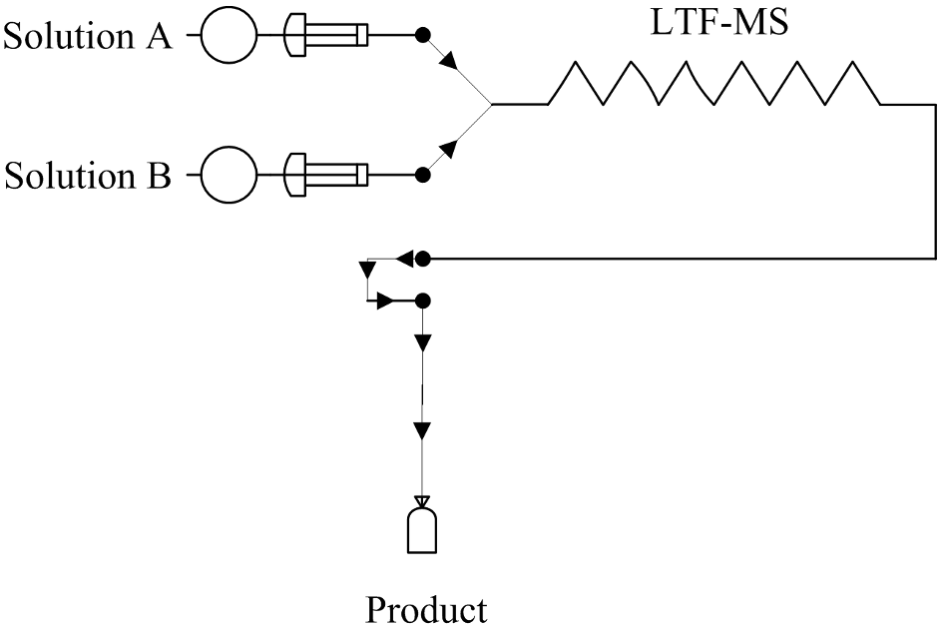
Schematic representation of the microreactor set up.

Using two (1 mL) SGE glass syringes and PTFE tubing of 0.5 mm internal diameter, the reactants (diazonium salt and azo coupling component solutions) were fed to an LTF-MS (Volume: 0.2 mL, channel size: 1 mm, geometry: 115 × 60 × 6 mm) microreactor plate (Figure 6). The microreactor plate was dipped into a temperature control bath and delivery of the reactants to the plates was enabled by two Chemyx fusion 100 classic syringe pumps.

### Preparation of reactant solutions

Solution A (diazotized primary aromatic amine): 2,4-dimethylaniline (0.2918 g) was dissolved in approximately 0.8 mL of concentrated 32% HCl and cooled. To this cooled solution, 3 mL of cold sodium nitrite solution (0.29 g in 5 mL of distilled water) was added drop wise until the potassium starch iodide paper test was positive. DMF (20 mL) was added to this, after which the solution was made up to a volume (100 mL) with distilled water.

Solution B (coupler): 2-naphthol (0.35 g) was dissolved in 10% aqueous NaOH (10 mL) to which DMF (15 mL) was added. The pH of the solution was buffered to the appropriate pH intended for the investigation (pH 5.11, 7.39 and 10.83). The solution was then made up to a volume (50 mL) with distilled water.

Similarly, the reactant solutions A and B (diazotized primary aromatic amine and coupler) in the azo coupling of diazonium salt solution of *p*-nitroaniline to diphenylamine were prepared as follows:

Solution A (diazotized primary aromatic amine): *p*-nitroaniline (0.2918 g) was dissolved in hot concentrated 32% HCl (0.8 mL) and cooled. To this cooled solution, cold sodium nitrite solution (0.29 g in 5 mL of distilled water) was added drop wise until the potassium starch iodide paper test was positive. DMF (20 mL) was added to this after which the pH of the solution was buffered to afford the various working pH intended for the study (pH: 3.5, 5.66 and 6.94). The solution was then made up to a volume (100 mL) with distilled water.

Solution B (coupler): diphenylamine (0.35 g) was dissolved in methanol (50 mL).

### Azo coupling reactions in microreactors

A central composite experimental design with a total of 12 experiments was used for each of the optimization studies at the various pH levels for the two reactions. The experiments were performed in a randomized manner. In addition, the flow rate of the solutions A and B was also varied as is shown in Table 9 below. The temperature of the batch diazotization reaction was kept constant at 0 °C while that of the azo coupling reaction.

**Table 9:** Experimental domain.

Reaction parameters	Minimum	Maximum

Flow rate (mL/min)	0.03	0.7
Temperature (°C)	0	50

### Microreactor set up; continuous flow synthesis of azo compounds

**Figure 7:**
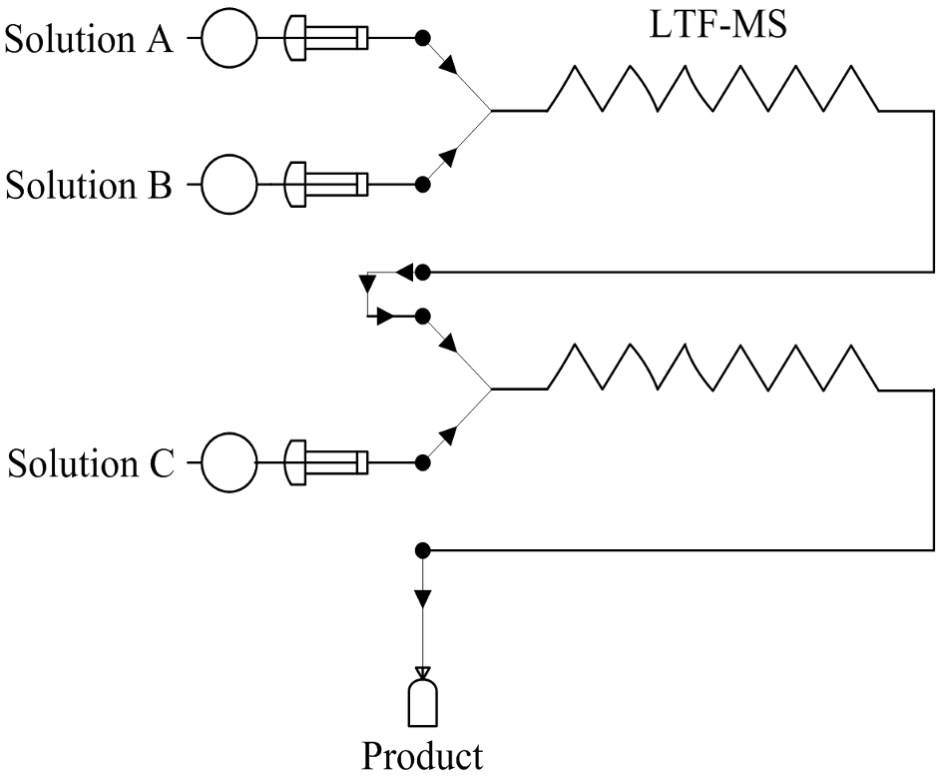
Schematic representation of the microreactor set up.

Using three (5 mL) SGE glass syringes and PTFE tubing (i.d. 0.5 mm, length: 340.2 mm connecting from the first reactor plate to the second reactor plate and 380.7 mm connecting from the second reactor plate to the sample collection bottle) reactant solutions A (amine + HCl solution), B (sodium nitrite solution) and C (coupler) were fed into two LTF-MS microreactor plates (reactor volume: 0.2 mL, channel size: 1 mm, geometry: 115 × 60 × 6 mm) joined by PTFE tubing (i.d. 0.5 mm). The delivery of the reactants was enabled by three Chemyx Fusion 100 classic syringe pumps as shown in Figure 7. The reaction temperature for the diazotization (0 °C) and azo coupling (25 °C) reactions was maintained with the aid of an ice and water bath respectively.

### Preparation of reactant solutions

Solution A (amine + HCl solution): 2,4-dimethylaniline (0.2918 g) was dissolved in approximately 0.8 mL of concentrated 32% HCl. DMF (20 mL) was added to this, after which the solution was made up to a volume (100 mL) with distilled water.

Solution B (sodium nitrite solution): Sodium nitrite (0.2914 g) was dissolved in DMF (5 mL) and made up to a volume (50 mL) with distilled water.

Solution C (coupler): 2-naphthol (0.35 g) was dissolved in 10% aqueous NaOH (10 mL) to which DMF (15 mL) was added. The pH of the solution was adjusted to 8.55–9 with 10% glycine solution. The solution was then made up to a volume (50 mL) with distilled water.

For the azo coupling reactions performed in acidic reaction media, the reactant solution C was prepared as follows.

Solution C (coupler): 1-naphthylamine (0.3477 g) was dissolved in glacial acetic acid (10 mL of 10% glacial acetic acid solution) and buffered to a pH of approximately 5.77–6. The solution was then made up to volume (50 mL) with distilled water.

### Microreactor diazotization and consequent azo coupling reactions

A central composite experimental design with a total of 20 experiments was used for this optimization study, where the diazotization of 2,4-dimethylaniline and its in situ azo coupling to 2-naphthol was used as a model reaction. The experiments were performed in a randomized manner. The temperature of the diazotization reaction was kept constant at 0 °C while that of the azo coupling reaction was kept at 25 °C (Table 10).

**Table 10:** Experimental domain.

Reaction parameters	Minimum	Maximum

HCl + amine (mL/min)	0.09	0.3
Sodium nitrite (mL/min)	0.01	0.05
Coupler (mL/min)	0.01	0.12

### Scaled up microreactor diameter set up: PTFE tubing i.d. 1.5 mm

**Figure 8:**
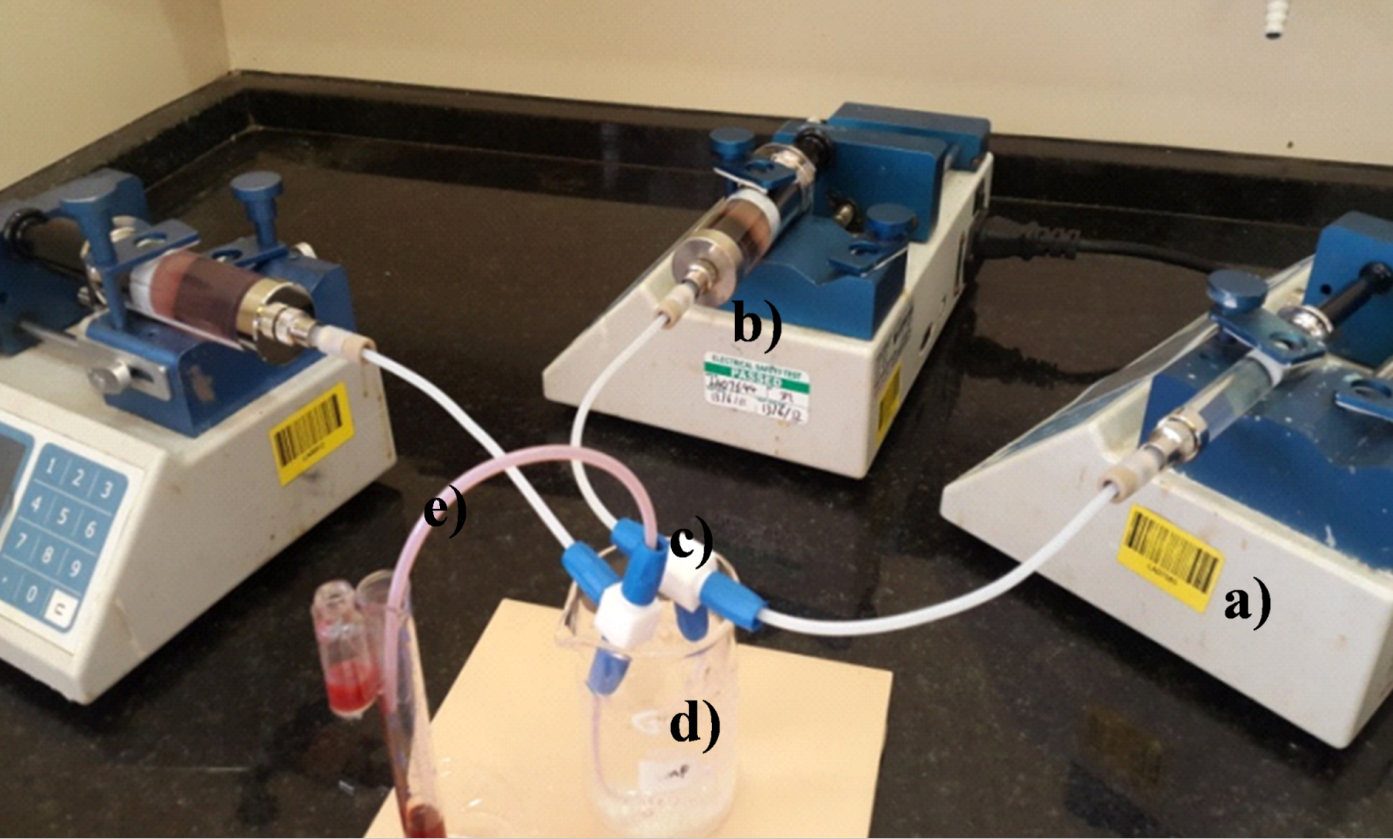
Scaled up microreactor set up: PTFE tubing i.d. 1.5 mm a) Chemyx Fusion 100 classic syringe pump, b) SGE glass syringe, c) T-mixer, d) Ice bath: Diazotization reaction and e) Room temperature: Azo coupling reaction.

Keeping the residence time established in the optimization reactions constant, the scale up was carried out in PTFE tubing (i.d. 1.5 mm, length: 150.93 mm and 155.43 mm for the diazotization and azo coupling reactions, respectively). Using three SGE glass syringes and two 3-Way-Tee mixers (Omnifit labware, Pore size: 8.0 mm i.d., 0.5-4 mm OD), the delivery of reactant solutions A (amine + HCl solution), B (sodium nitrite solution) and C (coupler) into the PTFE tubing (i.d. 1.5 mm) was enabled by three Chemyx Fusion 100 classic syringe pumps (Figure 8). The reaction temperature for the diazotization reaction was kept at 0 °C with the aid of ice. The azo coupling reaction was performed at room temperature (25 °C).

### Sample preparation

The microreactor set up was stabilized for 10 minutes between each experiment. In order to obtain substantial amount of samples for analysis, they were collected for a period of 1 minute each in a sample vial containing HCl (0.2 mL of 1 M). The mixture was then diluted with DMF (1 mL).

#### Sample analysis

Off-line reversed phase HPLC using a Phenomenex Luna 5 µ C18 100 A (250 × 4.60 mm × 5 microns) column under the following conditions; flow rate: 1.2 mL/min, mobile phase (acetonitrile 0.1% formic acid (75:25)) equipped with a variable wavelength detector was used for sample analysis. The external standard calibration HPLC method was used to quantify the amount of coupler utilized in the reaction. The wavelength used for quantification of the 2-naphthol was 349 nm.

#### Data analysis

The total volume of samples collected (tvs_collected_) was calculated by multiplying the total flow rate of the reactant solutions (tfr_ABC_) by the total sample collection time (*t*_collection_). The reaction time was calculated by dividing the total reaction space volume i.e. the total volume of the two LTF-MS plates, the PTFE tubing used to join the two mixers and also that leading to the final outlet: the point of sample collection by the total flow rate of reactant solutions (tfr_ABC_). For purposes of data analysis, all flow rates were converted to liters/minute.

## Supporting Information

File 1 Additional diagrams and NMR spectra.
