# Supplementary material for: The in situ generation and reactive quench of diazonium compounds in the synthesis of azo compounds in microreactors
Source: Beilstein J Org Chem. 2016 Sep 6;12:1987–2004. doi: 10.3762/bjoc.12.186 (PMC5082718; doi:10.3762/bjoc.12.186)
Supplement: File 1 — Additional diagrams and NMR spectra. [file Beilstein_J_Org_Chem-12-1987-s001.pdf]

Supporting Information

for

The in situ generation and reactive quench of diazonium compounds in the synthesis of azo compounds in microreactors

Faith M. Akwi and Paul Watts*

Address: Nelson Mandela Metropolitan University, University Way, Port Elizabeth, 6031, South Africa.

Email: Paul Watts - Paul.Watts@nmmu.ac.za

Additional diagrams and NMR spectra

Azo coupling reaction in the synthesis of Sudan II azo dye in Little Things Factory microreactors

Statistical Model validation for the predictive multiple regression model fitted in the investigation of reaction parameters, i.e., flow rate, temperature and pH which have an effect on the azo coupling reaction in the synthesis of Sudan II azo dye.

Predicted conversion (%) = - 42.4512 + 27.9443 * pH - 6.5854 * Flow rate + 0.3187 * Temperature - 1.6268 * pH^2 - 0.0061 * $Temperature^2$

It was also found that about 85% of the variation seen in the observed data can be explained by the model.

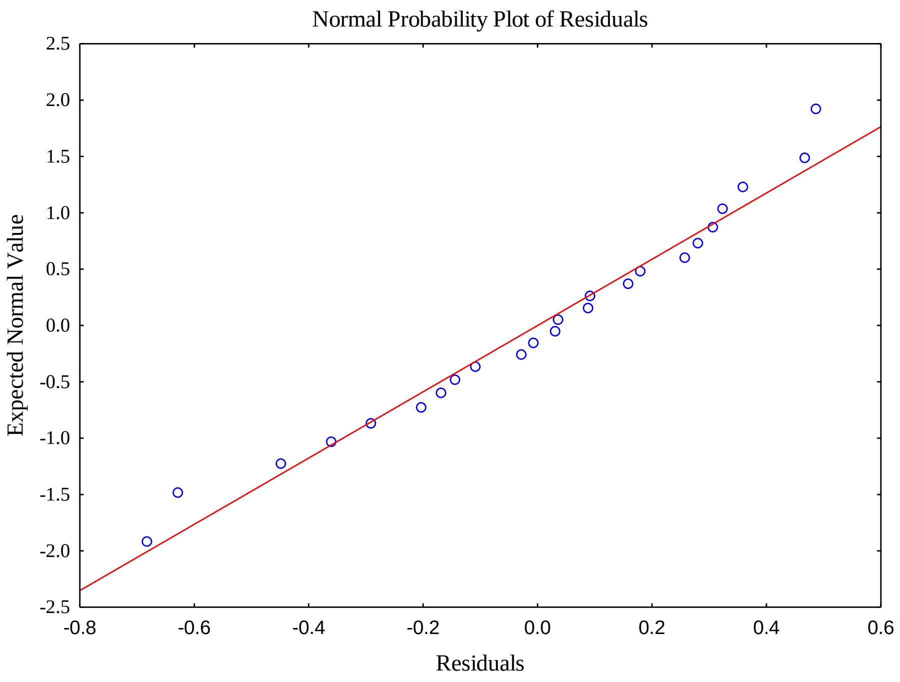

Figure S1: Normal probability plot.

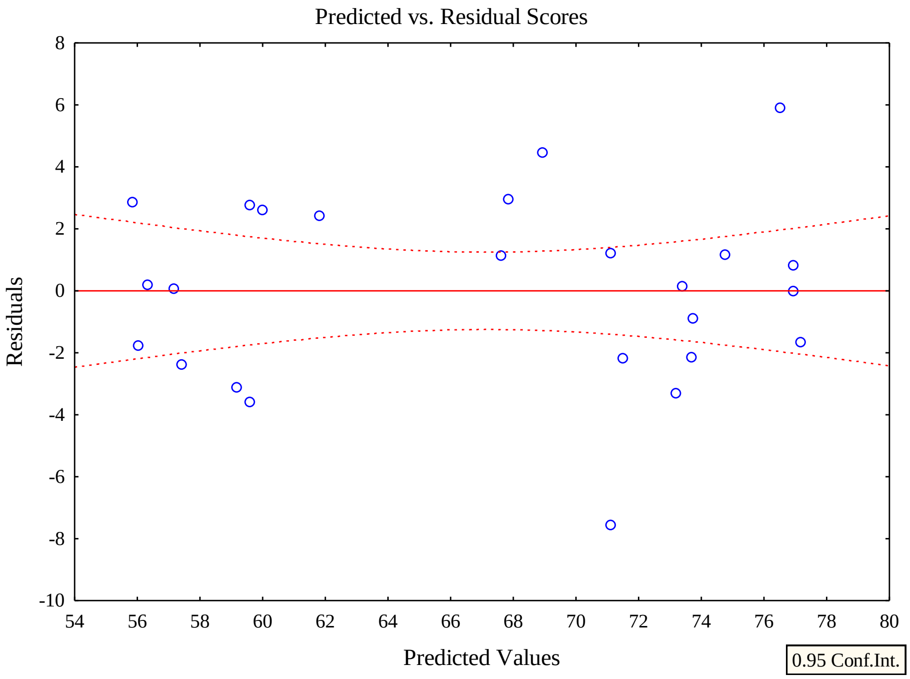

Figure S2: Scatter plot of predicted values vs. residual values

Azo coupling reaction in the synthesis of 4-(2-(4-nitrophenyl)diazenyl)-*N*-phenylbenzenamine in Little Things Factory microreactors

Statistical Model validation for the predictive multiple regression model fitted in the investigation of reaction parameters, i.e., flow rate, temperature and pH which have an effect on the azo coupling reaction in the synthesis of 4-(2-(4-nitrophenyl)diazenyl)-*N*-phenylbenzenamine in LTF-MS microreactors

Model fitting was performed using statistica-12 Statsoft program where a Logit model transformation was carried out on the response which in this case was the percentage conversion. Three outliers (Cases 18, 24 and 25) were removed from the raw data set.

The predictive multiple regression model obtained was as follows;

Predicted conversion (%) = 8.14853 - 4.48258 * pH + 0.01204 * Temperature + 0.61041 * Flow rate + 0.43744 * pH^2

It was also found that about 75% of the variation seen in the observed data can be explained by the model.

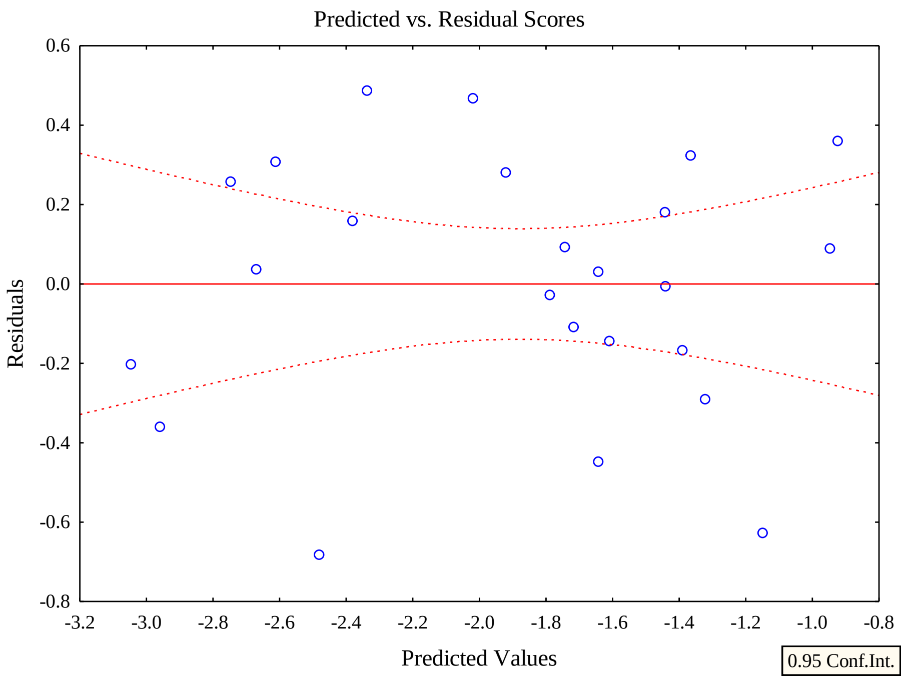

Figure S3: Scatter plot of predicted values vs residual values.

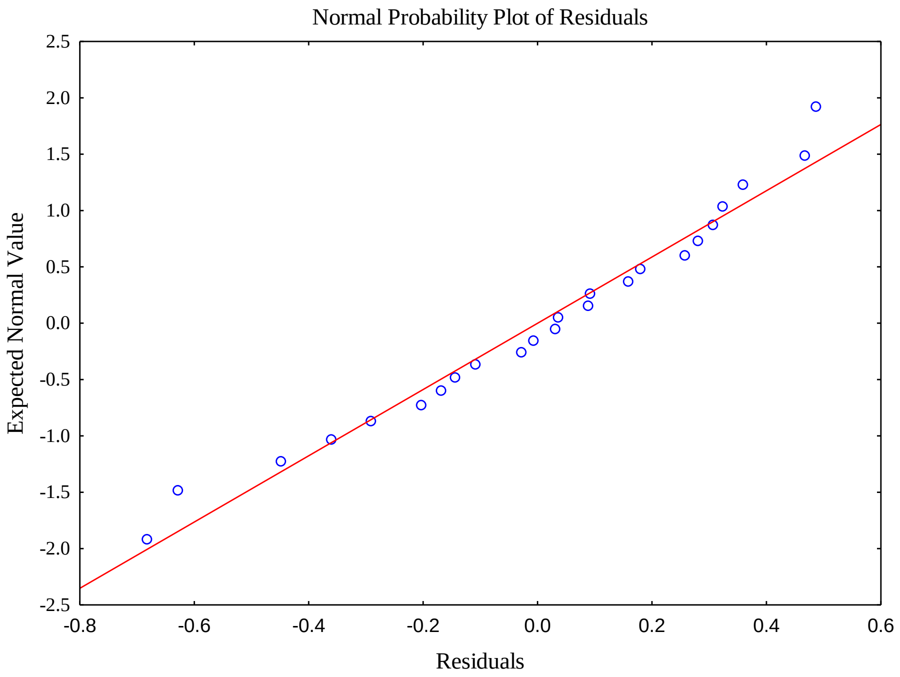

Figure S4: Normal probability plot.

Continuous flow synthesis of Sudan II azo dye in LTF-MS microreactors

Table S1: Summary of statistical multiple regression data analysis

	Regression Summary for Dependent Variable: Conversion % $R= .54875331$ $R^2= .30113019$ Adjusted $R^2= .17009210$					
N=20	b*	Std.Err.	b	Std.Err.	t(16)	p-value
Intercept			96.2480	1.83096	52.56701	0.000000
Amine + HCl (l/min)	0.387552	0.208997	11.3725	6.13290	1.85435	0.082215
Sodium Nitrite (l/min)	-0.072019	0.208996	-12.5000	36.27452	-0.34459	0.734887
Coupler (l/min)	-0.382741	0.208997	-21.0237	11.48002	-1.83133	0.085737

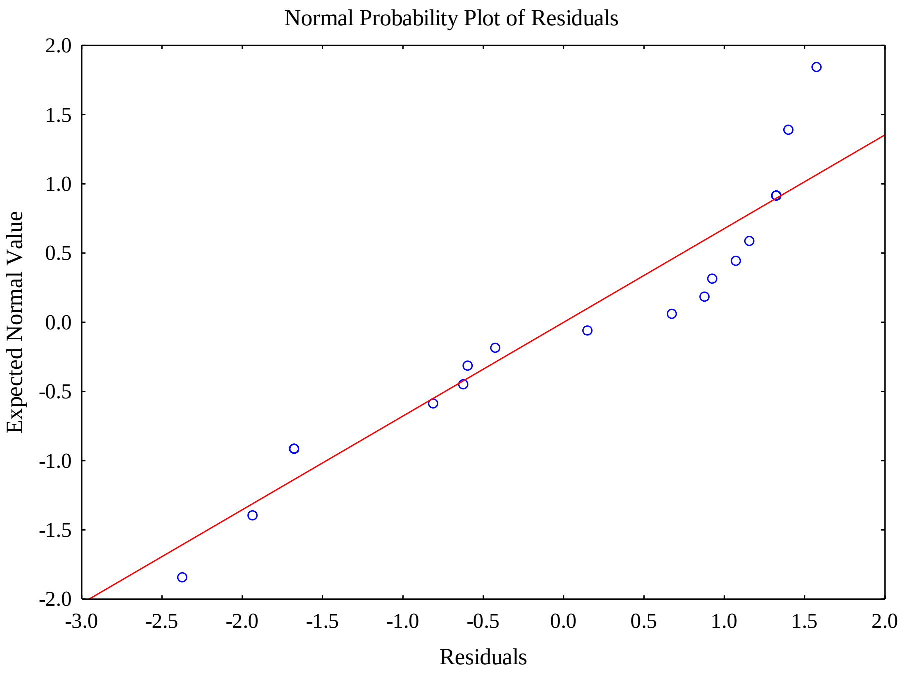

Figure S5: Normal probability plot.

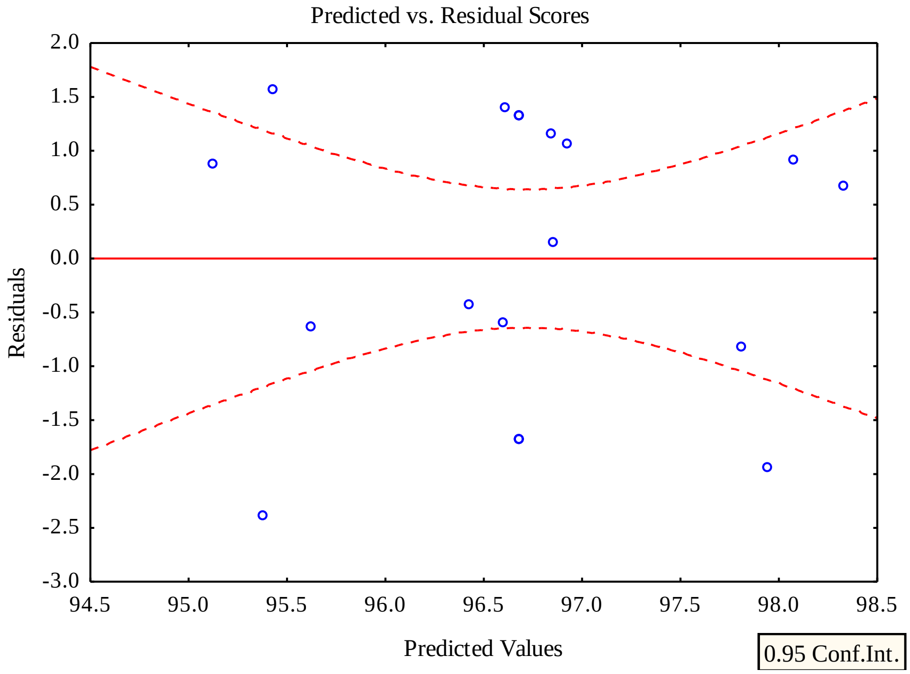

Figure S6: Scatter plot of predicted values vs residual values.

^{1}H and ^{13}C NMR spectra of principle compounds synthesized from the model reactions used in the optimized synthesis of azo compounds in continuous flow systems.

Compound: 1-((2,4-dimethylphenyl)azo)naphthalen-2-ol

^{1}H NMR spectra

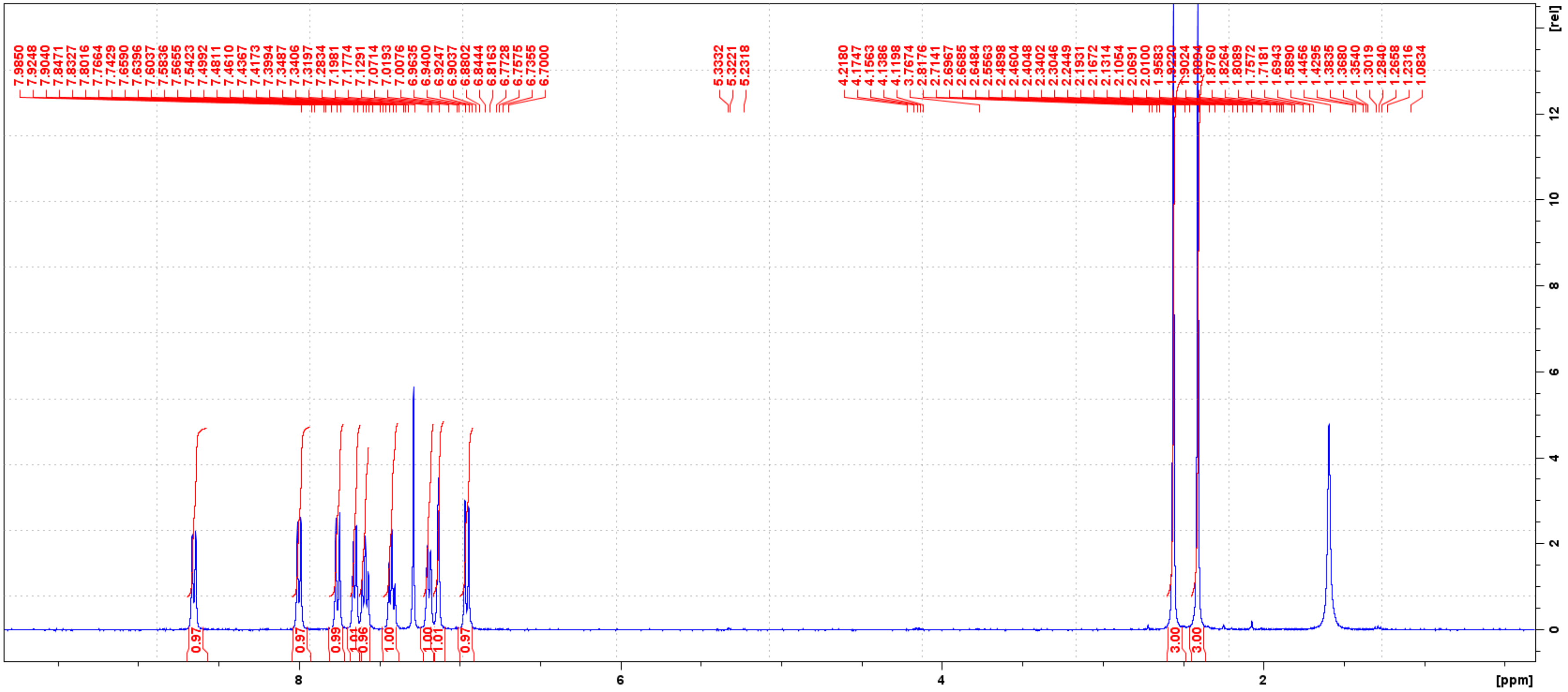

^{13}C NMR spectra

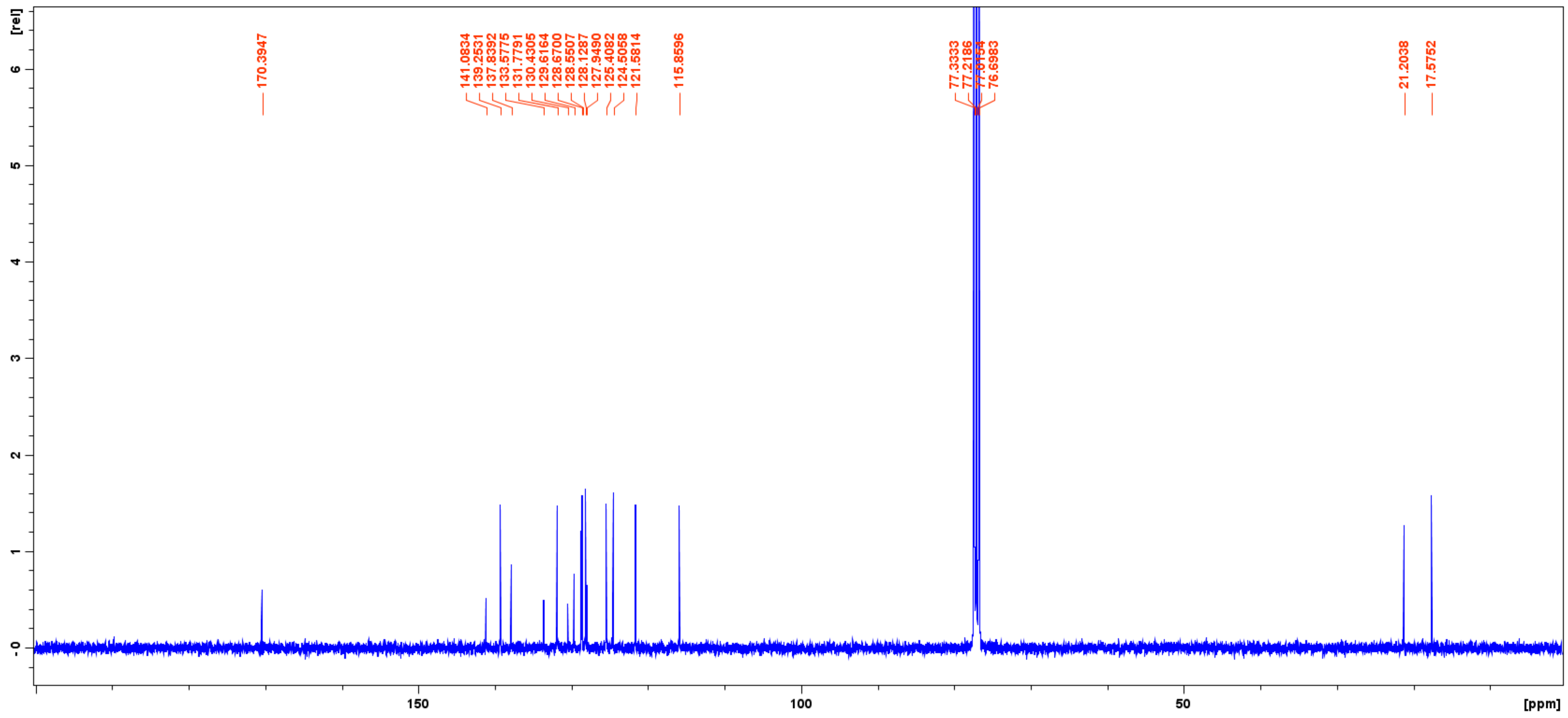

Compound: 4-(2-(4-nitrophenyl)diazenyl)-*N*-phenylbenzenamine

^{1}H NMR spectra

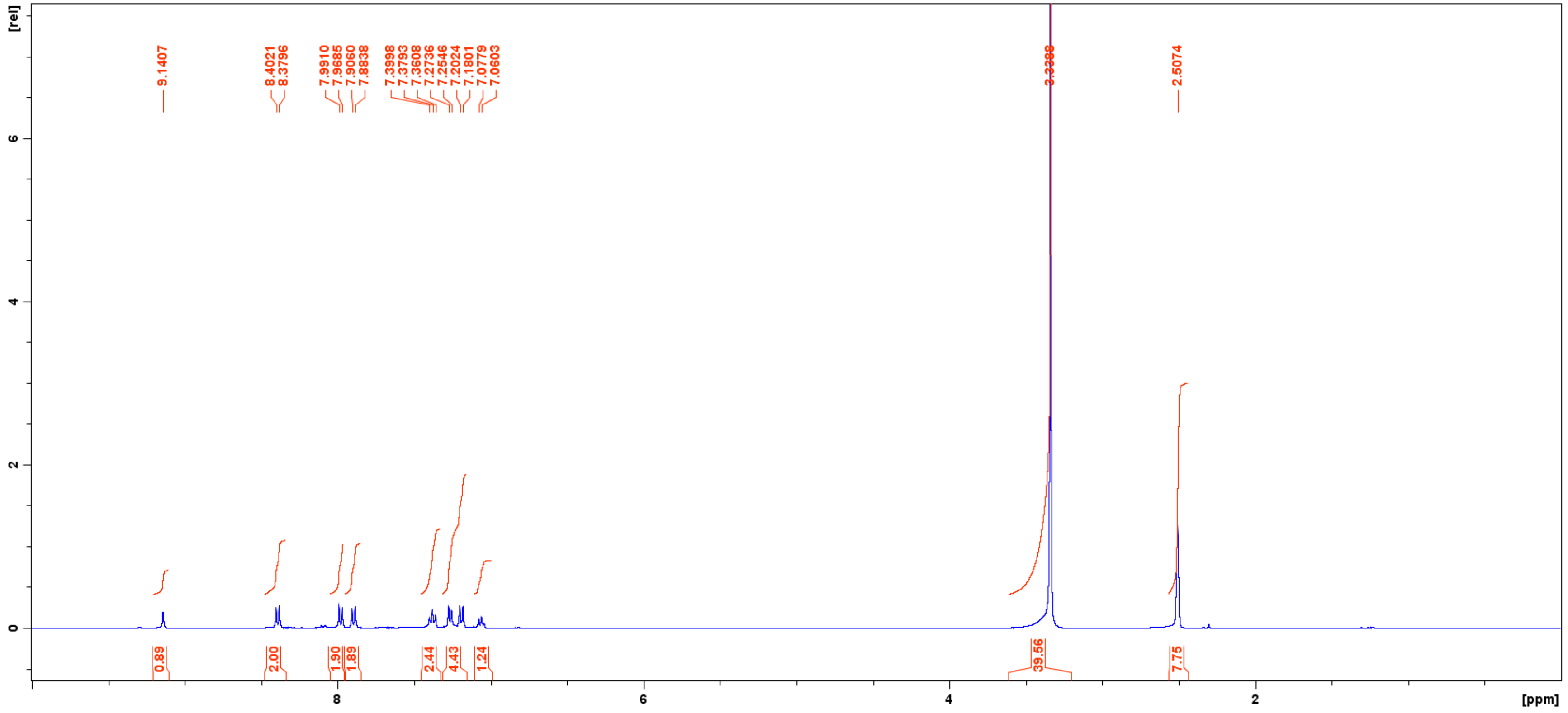

^{13}C NMR spectra

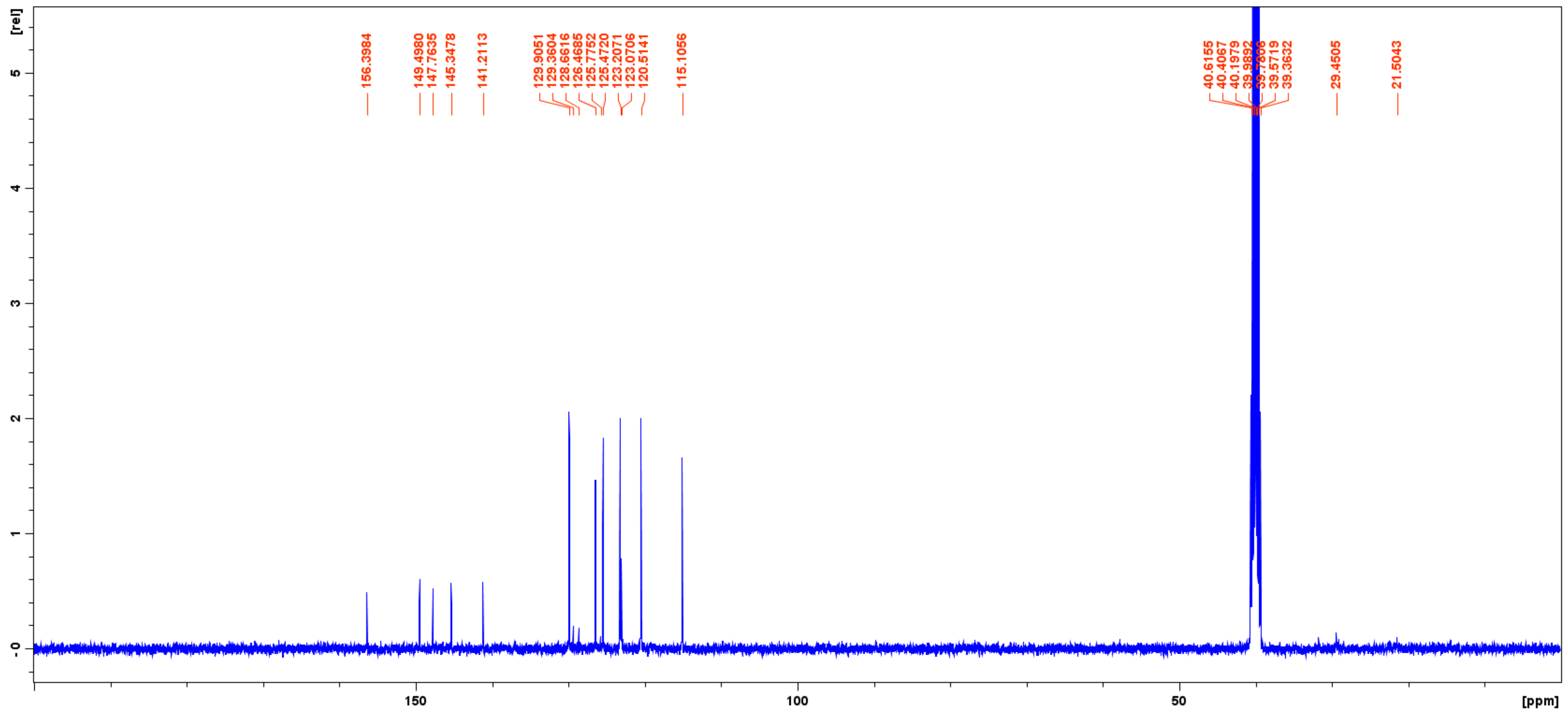

^{1}H and ^{13}C NMR spectra of selected azo compounds as shown in Table 9 of this paper

Compound Entry: 1

^{1}H NMR spectra

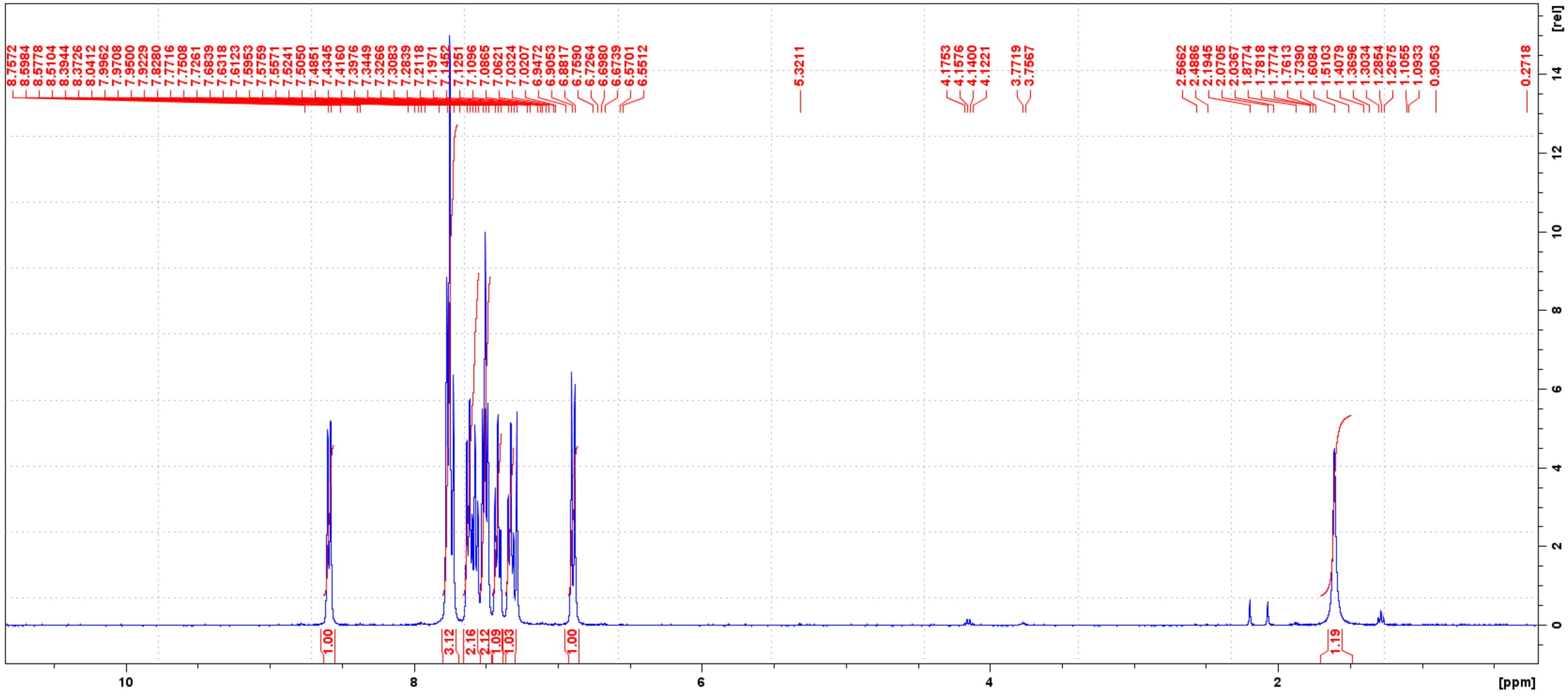

^{13}C NMR spectra

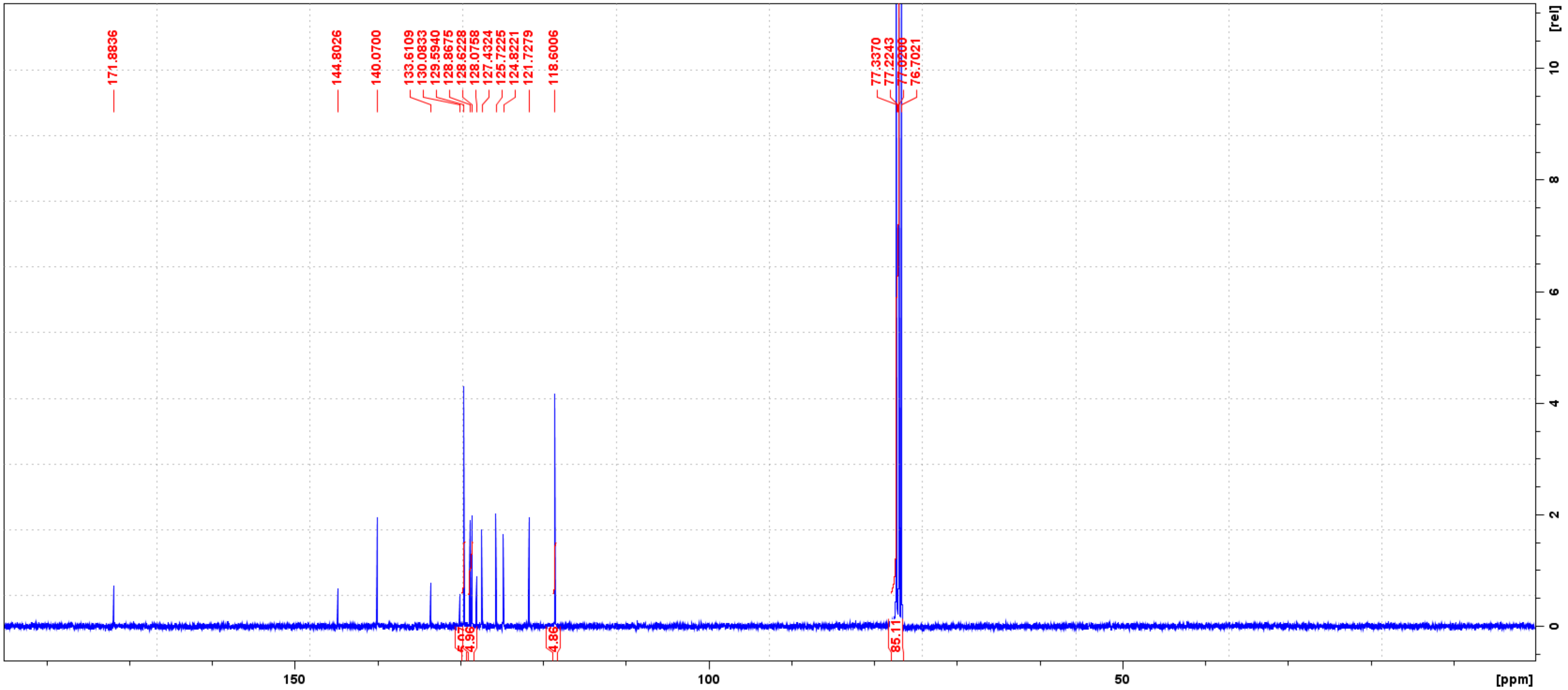

Compound Entry: 4

^{1}H NMR spectra

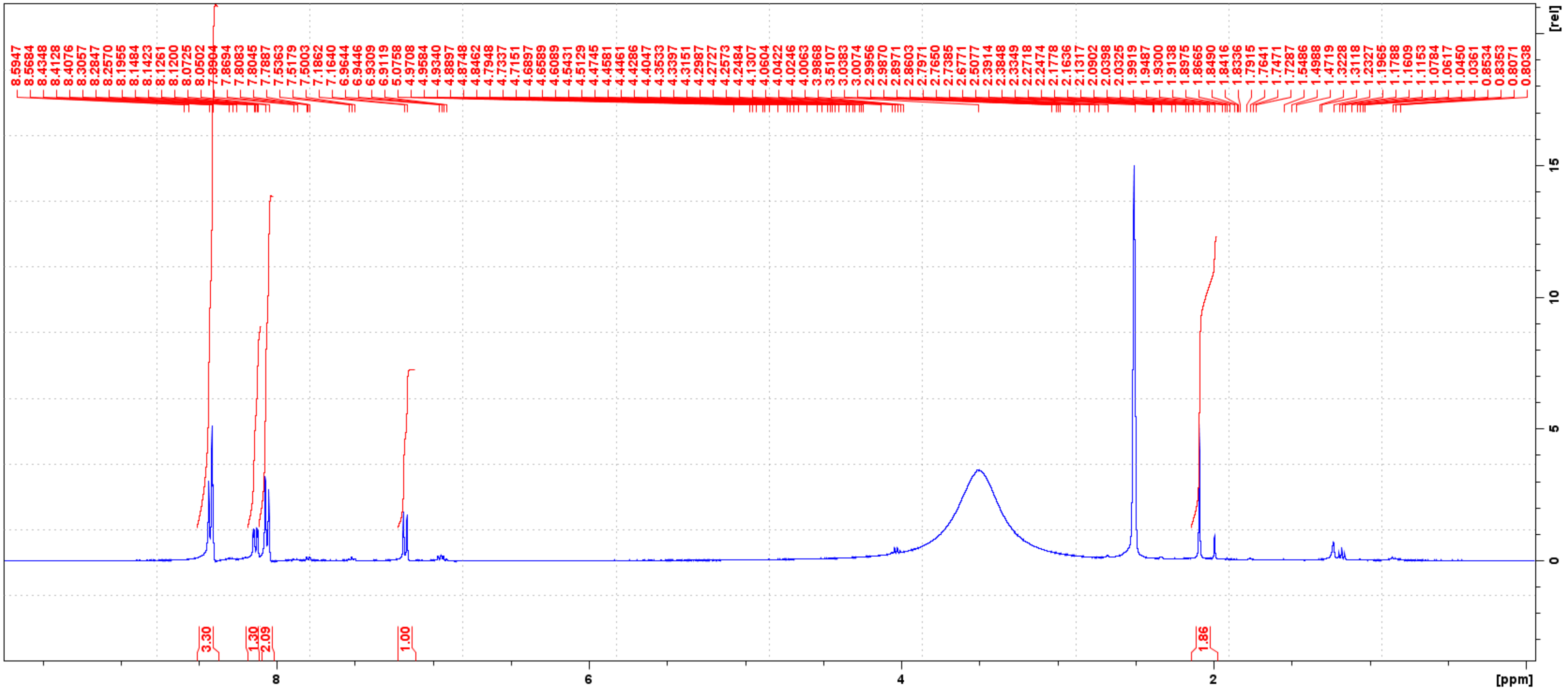

^{13}C NMR spectra

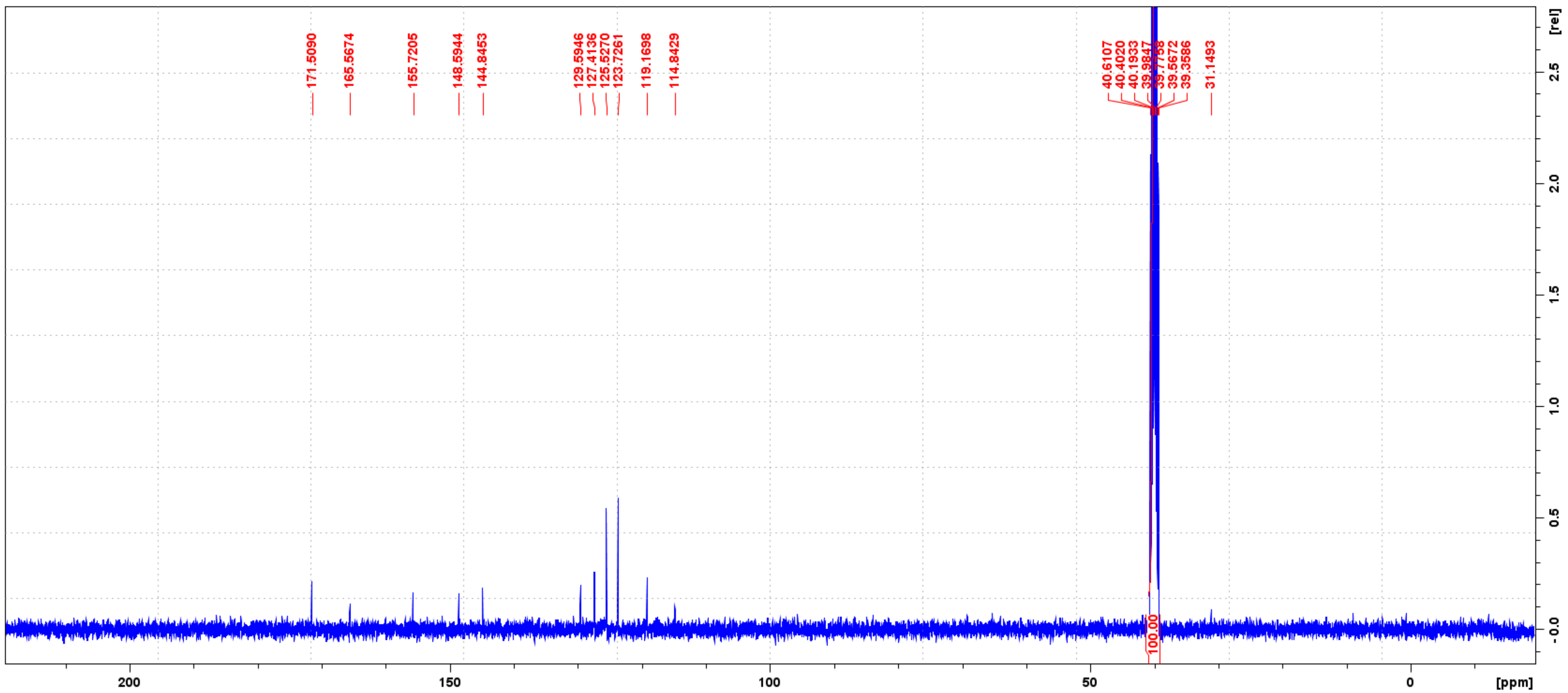

Compound Entry: 7

^{1}H NMR spectra

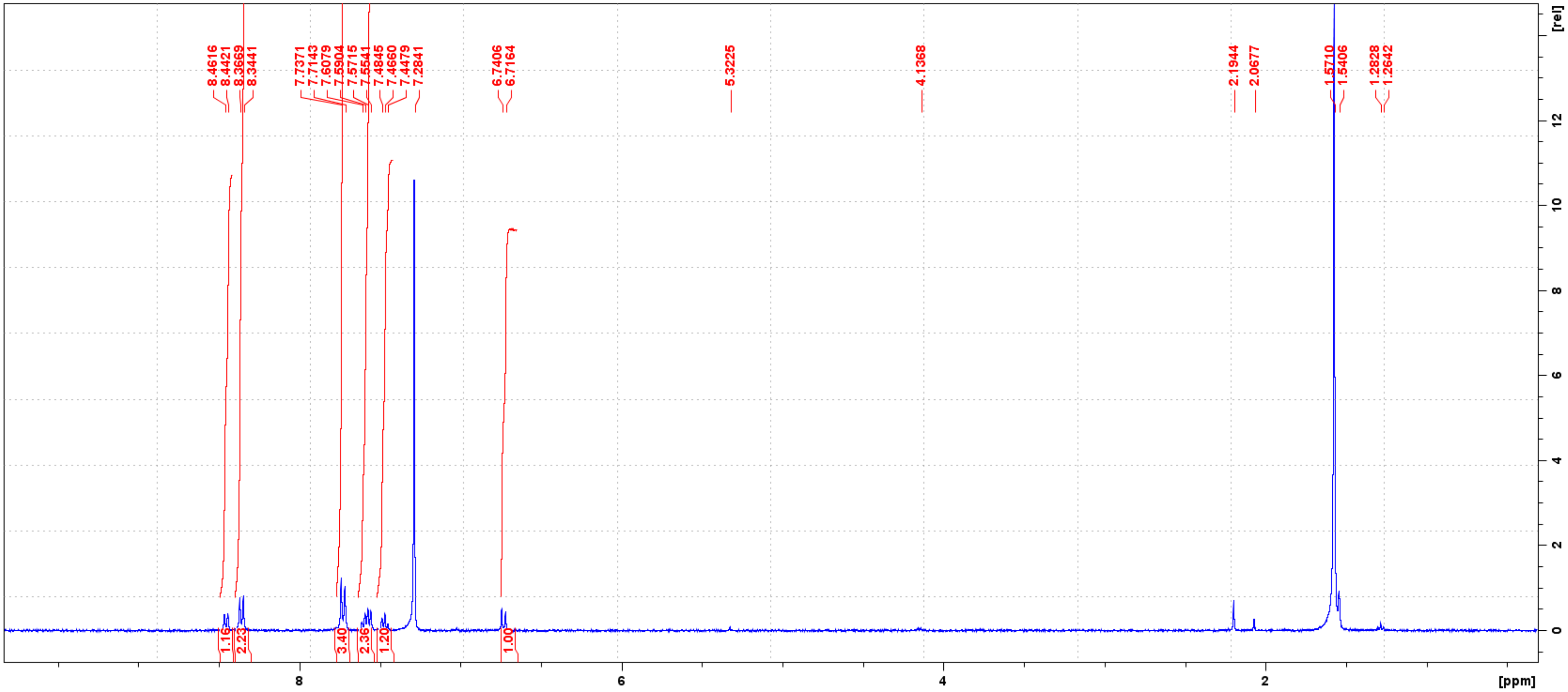

^{13}C NMR spectra

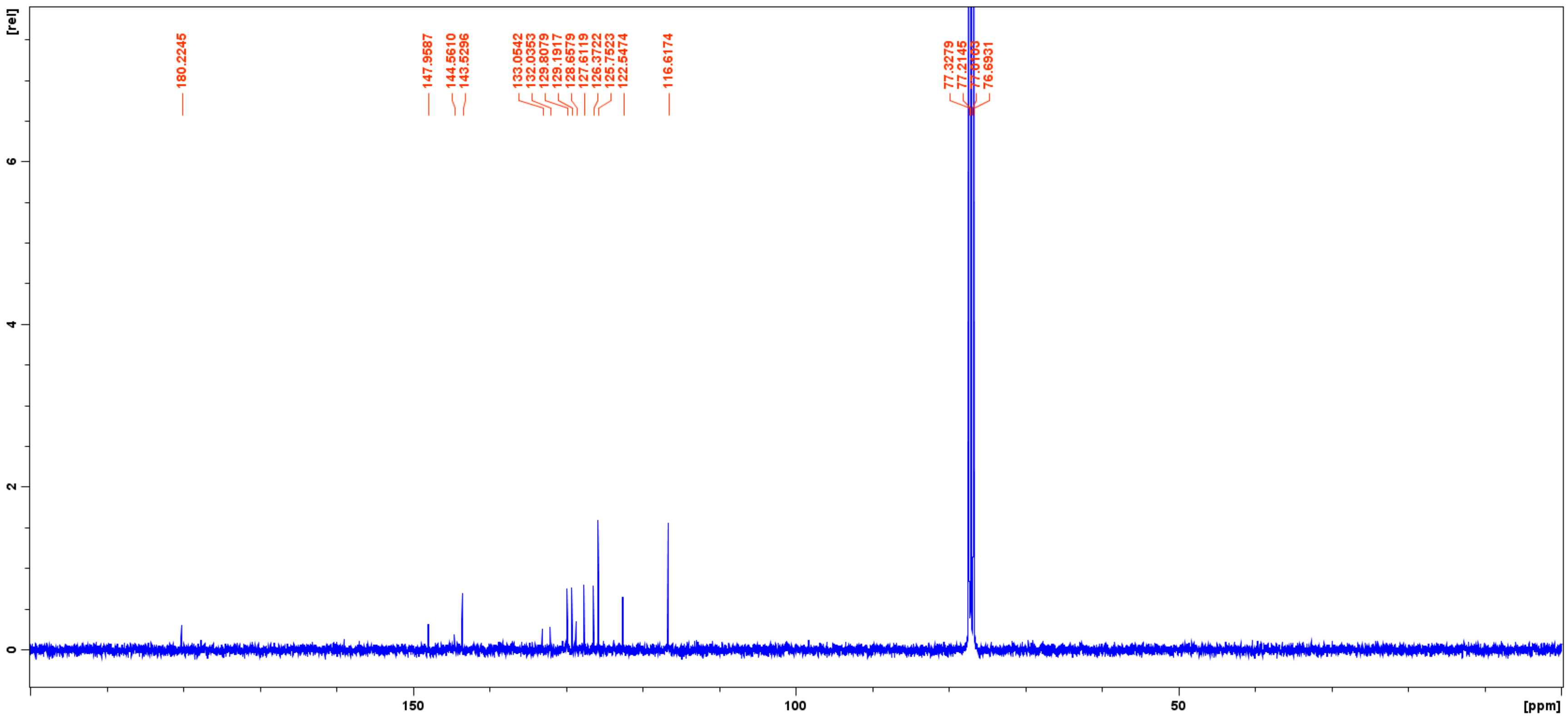

Compound Entry: 9

^{1}H NMR spectra

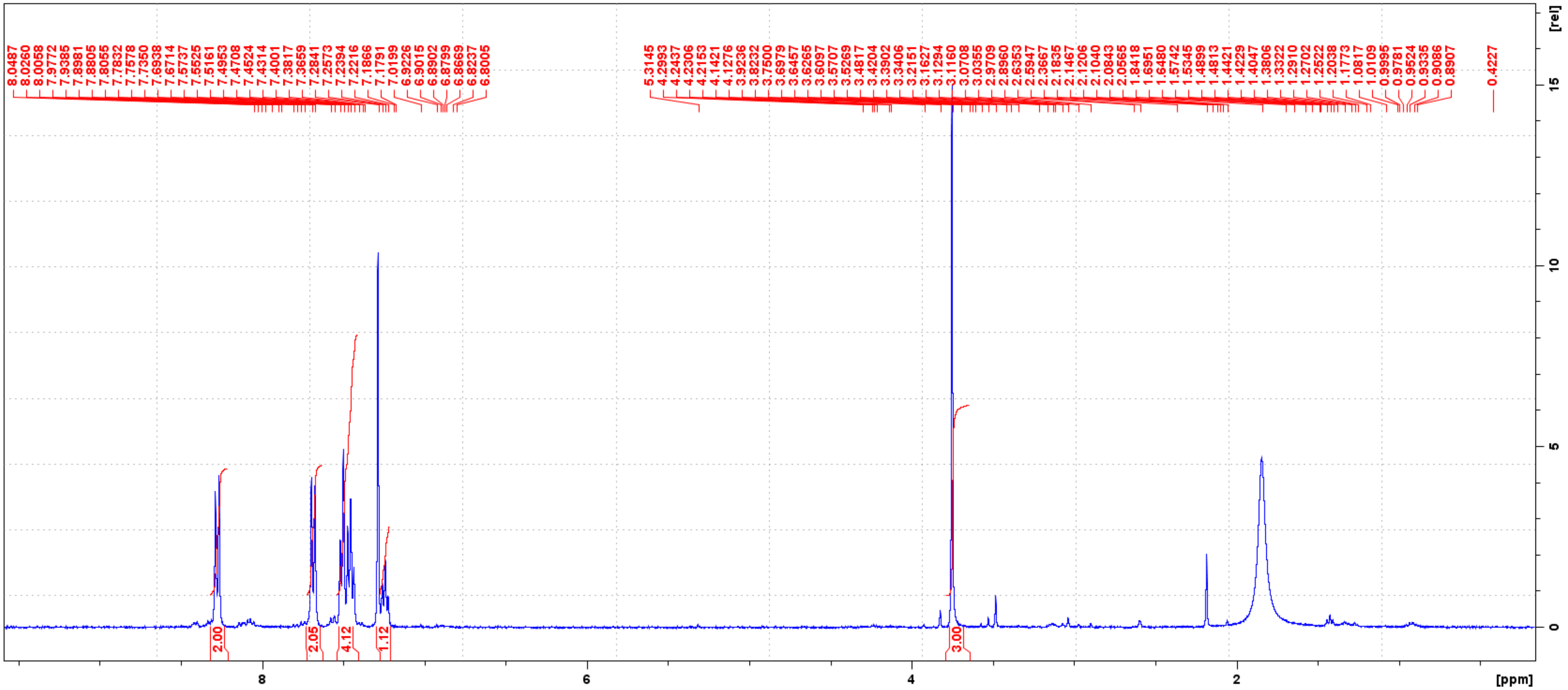

^{13}C NMR spectra

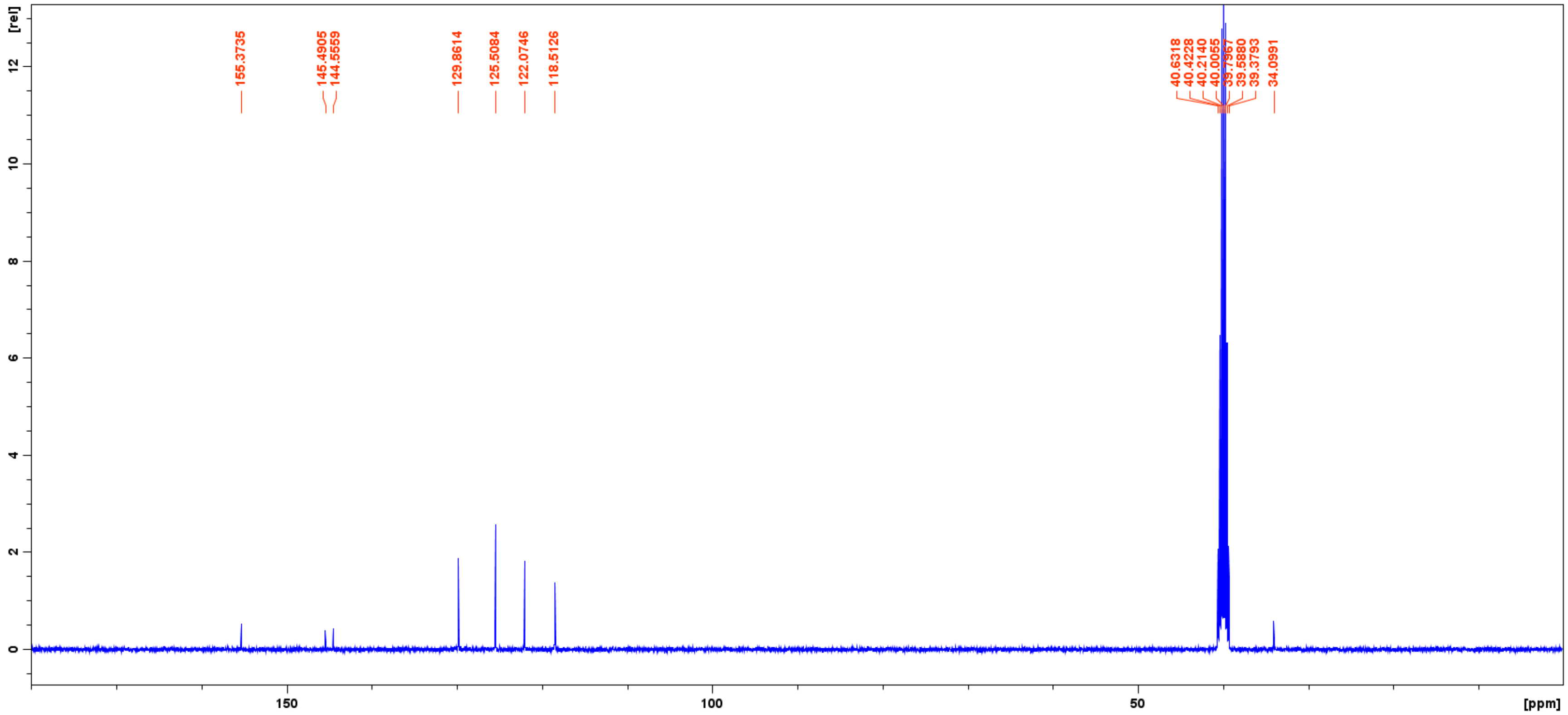

Other reactions were only followed by HPLC by monitoring consumption of the relevant starting materials.
